# In vitro cobalt stress alters *Adhatoda vasica* anatomy, antioxidant defense, and metabolite profiles with docking insights

**DOI:** 10.1186/s12896-025-01088-9

**Published:** 2025-12-27

**Authors:** Ahmed M. Zaher, Fatma A. Al-Kahtany, Ahmed A. K. Mohammed, Fatma A. Farghaly, Abeer A. Radi, Afaf M. Hamada

**Affiliations:** 1 Pharmacognosy Department, Faculty of Pharmacy, Assiut University, Assiut, 71515 Egypt; 2 Biology Department, Faculty of Science, Ibb University, Ibb, Yemen; 3 Chemistry Department, Faculty of Science, Assiut University, Assiut, 71516 Egypt; 4 Botany and Microbiology Department, Faculty of Science, Assiut University, Assiut , 71516 Egypt

**Keywords:** *Adhatoda vasica* L., Antioxidant enzymes, Cobalt, Anatomy, Metabolite profiling, Molecular docking

## Abstract

The growing demand for medicinal plants in herbal medicine highlights their therapeutic value, yet heavy metal contamination, such as cobalt (Co), poses potential health risks. Cobalt’s dual role as an essential micronutrient and a toxic pollutant necessitates a more profound understanding of its impact on medicinal plants like *Adhatoda vasica*. We investigated how varying Co concentrations affect *A. vasica* shoot growth, leaf anatomy, antioxidant enzyme activity, and secondary metabolite profiles. Additionally, molecular docking was performed to assess the interaction of the metabolites with the skin cancer-related protein anti-ssDNA antigen-binding fragment (PDB code: 1P7K). Low Co concentrations (50 µM) enhanced shoot dry weight by 41.45%, while higher levels (100–1000 µM) reduced it by up to 66.86%. Cobalt exposure increased hydrogen peroxide (H_2_O_2_) and lipoxygenase (LOX) activity, indicating reactive oxygen species (ROS)-induced oxidative stress. Higher Co levels increased superoxide dismutase (SOD), catalase (CAT), phenylalanine ammonia-lyase (PAL), and polyphenol oxidase (PPO) but decreased peroxidase (POD) and ascorbate peroxidase (APX) activity. HPLC-UV and GC-MS data showed that Co altered the secondary metabolites of *A. vasica*, including phenolics, flavonoids, alkaloids, and terpenoids, in both qualitative and quantitative ways. Molecular docking shows that naringin has a higher binding affinity (-9.2 kcal/mol) to PDB: 1P7K than phenolics (-4.8 to -6.4 kcal/mol). Cobalt stress significantly impacts *A. vasica*, altering its leaf structure, growth, and antioxidant defenses. These effects extend to secondary metabolites in a dose-dependent manner. These findings highlight the plant’s potential for Co tolerance and its metabolites’ therapeutic promise, particularly naringin, for skin cancer applications.

**Supplementary Information:**

The online version contains supplementary material available at 10.1186/s12896-025-01088-9.

## Introduction

Heavy metal contamination, driven by industrial activities such as mining, oil refining, and waste disposal, poses a global threat by entering the food chain and affecting ecosystems [1, 2]. Cobalt (Co), a trace element, exemplifies this challenge due to its dual role as an essential micronutrient for plants and a potential toxicant at higher concentrations [3]. Naturally, soil Co levels range from 8 to 25 mg kg⁻¹, with agricultural regulations capping permissible levels at 20 mg kg⁻¹ [4]. While plants thrive at low Co concentrations (< 0.08 mg kg⁻¹ soil), levels up to 40 ppm can impair growth, and groundwater Co varies from 0.006 to 0.43 mg L⁻¹ [5–7]. The International Agency for Research on Cancer (IARC) classifies Co as a potential human carcinogen, underscoring its health risks [8].

As a transition metal, Co catalyzes Fenton reactions, generating reactive oxygen species (ROS) that cause lipid peroxidation, membrane disruption, enzyme deactivation, and photosynthetic decline [9, 10]. Plants combat these effects using enzymatic and non-enzymatic defense systems [7, 11, 12]. The phenylpropanoid and shikimate pathways, activated under stress, produce secondary metabolites like phenolics and flavonoids, which chelate metals and scavenge ROS, enhancing plant tolerance [13, 14]. These metabolites also hold industrial and medicinal value, contributing to plant–environment interactions [15, 16]. However, heavy metal uptake and transport depend on factors like plant species, soil properties, and organ anatomy, with leaf anatomical changes often overlooked despite their role in metal sequestration and tolerance [17].

*Adhatoda vasica*, a medicinal shrub of the Acanthaceae family, is valued in Unani and Ayurvedic medicine for its therapeutic properties and is recognized by the World Health Organization for primary healthcare applications [18]. Despite its widespread use, the impact of heavy metals on medicinal plants like *A. vasica* remains underexplored compared to crop species, raising concerns about contamination risks in herbal remedies [19, 20]. *Adhatoda vasica* is recognized for several medicinal applications; however, its efficacy against skin cancer, specifically the mechanistic details involving metabolite-protein interactions, has yet to be explored.

This study addresses these gaps by examining how Co stress affects *A. vasica* shoot growth, leaf anatomy, antioxidant defenses, and secondary metabolite profiles. We hypothesize that Co induces dose-dependent anatomical and biochemical changes, influencing ROS management and metabolite production. Additionally, we employ molecular docking to explore interactions between *A. vasica* metabolites (alkaloids, phenolics, and flavonoids) and the skin cancer-related protein anti-ssDNA antigen-binding fragment (PDB code: 1P7K), aiming to uncover therapeutic potential. Our findings clarify how *Adhatoda vasica* tolerates Co, which is essential for advancing its application in phytoremediation and exploring its value for drug development.

## Materials and methods

### Chemicals

All chemicals were sourced from Sigma-Aldrich (St. Louis, MO, USA). HPLC- and GC-MS-grade organic solvents were obtained from Thermo Fisher Scientific (Hanover Park, IL, USA).

### Plant growth

Shoot tips of *Adhatoda vasica* were collected from healthy plants at Assiut University’s Botany and Microbiology farm, Egypt (27°11′00″N, 31°10′00″E). After rinsing under running tap water for 20–30 min, shoot tips were surface-sterilized in a laminar flow hood by washing three times with sterile distilled water, soaking in 10% sodium hypochlorite for 10 min, and rinsing three times with sterile distilled water. Three sterilized shoot tips were aseptically placed in 195-mL culture jars containing 30 mL of sterile Murashige and Skoog (MS) medium [21], supplemented with 3 mg L⁻¹ 6-benzylaminopurine, 1 mg L⁻¹ α-naphthalene acetic acid, 4.4 g L⁻¹ MS salts, and 3% sucrose. The medium was adjusted to pH 5.7, solidified with 3 g L⁻¹ gelrite, and autoclaved at 121 °C for 15 min. Cobalt chloride (CoCl₂·6 H₂O) was added to the MS medium at concentrations of 0, 50, 100, 200, 400, 600, and 1000 µM [22]. The selected concentrations likely span from sub-toxic to potentially toxic levels to study both beneficial and adverse effects. For example, low concentrations (50–100 µM) might promote certain metabolic pathways, while higher ones (600–1000 µM) could induce stress or toxicity. Jars were incubated in a growth chamber under a 16/8 h light/dark photoperiod, 30 µmol m⁻² s⁻¹ light intensity, 25 ± 1 °C, and 50–60% relative humidity. Following 30 days, the proliferated shoots (defined as those with new growth) were harvested. Immediate fresh weight (FW) measurement was performed, and all samples were flash-frozen using liquid nitrogen and stored at -80 °C. The remaining shoots were oven-dried at 60 °C for 48 h to determine the dry weight (DW).

### Leaf anatomy

Leaf anatomy was analyzed to examine epidermal and vascular bundle structures. Leaves with prominent main veins were transversely sectioned into 7 μm slices using an optical microtome. Sections were stained with safranin for 30 min, followed by dehydration through a graded ethanol series (50–100%). They were then counter-stained with fast green for 30 s, washed with 95% ethanol, and finally cleared in a 1:1 xylene-ethanol solution for 30 min. Microstructures were examined using an Olympus CX41 light microscope equipped with an Olympus SC30 U-TV1X-2 camera (Tokyo, Japan).

### Estimation of cobalt content

Dried shoot samples were powdered and digested in a 1:3:1 (v/v) mixture of 60% HClO₄, concentrated HNO₃, and H₂SO₄ [23]. Digested samples were diluted to 10 mL with distilled water. Cobalt concentrations were quantified using a Buck 210 VGP atomic absorption spectrophotometer (Buck Scientific, USA), with calibration against a cobalt standard curve and inclusion of blanks for quality control.

### Estimation of hydrogen peroxide content

Hydrogen peroxide (H₂O₂) levels were measured spectrophotometrically following Velikova et al. [24]. Frozen shoots were homogenized in 0.1% trichloroacetic acid (TCA), centrifuged at 12,000 rpm for 15 min at 4 °C, and the supernatant was mixed with 10 mM potassium phosphate buffer (PPB, pH 7.8) and 1 M potassium iodide. After a 20-min incubation, absorbance was measured at 390 nm using a spectrophotometer. Hydrogen peroxide concentrations were calculated using a standard curve and expressed as mg g⁻¹ FW.

### Enzyme extraction

Frozen shoots were ground to a fine powder in liquid nitrogen and homogenized in 100 mM PPB (pH 7.8) containing 0.1 mM ethylenediaminetetraacetic acid (EDTA) and 0.1 g polyvinylpyrrolidone (PVP). The homogenate was centrifuged at 18,000 rpm for 10 min at 4 °C, and the supernatant was used for enzyme activity assays. Protein content was quantified using the Bradford method [25].

### Estimation of lipoxygenase activity

Lipoxygenase (LOX, EC 1.13.11.12) activity was assayed per Minguez-Mosquera et al. [26]. A substrate solution of linoleic acid, Tween-20, and distilled water was adjusted to pH 9.0 with NaOH, then to pH 6.5 with HCl, and diluted to 100 mL with 100 mM PPB. Enzyme aliquots were added, and absorbance changes at 234 nm were measured. Lipoxygenase activity was expressed as milligrams of protein per minute (DA_234_ mg protein^− 1^ min^− 1^).

### Estimation of superoxide dismutase activity

Superoxide dismutase (SOD, EC 1.15.1.1) activity was determined using the epinephrine oxidation method [27]. The reaction mixture contained 0.05 M Na₂CO₃ buffer (pH 10.2), 0.1 mL EDTA, enzyme aliquots, and epinephrine. Absorbance changes at 480 nm were measured, and SOD activity was expressed as milligrams of protein per minute (DA_480_ mg protein^− 1^ min^− 1^).

### Estimation of catalase activity

The activity of catalase (CAT, EC 1.11.1.6) was assessed by tracking H_2_​O_2_​ consumption [28]. The reaction mixture included 50 mM PPB (pH 7.0), 10 mM H₂O₂, and enzyme aliquots. Absorbance changes at 240 nm were recorded for 1 min, and CAT activity was expressed as milligrams of protein per minute (DA_240_ mg protein^− 1^ min^− 1^).

### Estimation of peroxidase activity

Peroxidase (POD, EC 1.11.1.7) activity was assessed using an enzymatic assay that monitored tetraguaiacol formation [29]. The reaction mixture contained 30 mM PPB (pH 7.0), 6.5 mM H₂O₂, 1.5 mM guaiacol, and enzyme aliquots. Absorbance changes at 470 nm were measured, and POD activity was expressed as milligrams of protein per minute (DA_470_ mg protein^− 1^ min^− 1^).

### Estimation of ascorbate peroxidase activity

Ascorbate peroxidase (APX, EC 1.11.1.11) activity was measured via ascorbic acid oxidation [30]. The reaction mixture included 50 mM PPB (pH 7.0), 0.1 mM EDTA, 1.2 mM H₂O₂, 0.5 mM ascorbic acid (AsA), and enzyme aliquots. Absorbance changes at 290 nm were recorded, and APX activity was expressed as milligrams of protein per minute (DA_290_ mg protein^− 1^ min^− 1^).

### Estimation of phenylalanine ammonia-lyase activity

Phenylalanine ammonia-lyase (PAL, EC 4.3.1.5) activity was determined by measuring trans-cinnamate production [31]. Enzyme aliquots were incubated in 50 mM borate buffer (pH 8.7) with phenylalanine at 37 °C for 1 h. The reaction was stopped with 0.5 N HCl, centrifuged at 2,000 rpm for 5 min, and absorbance was measured at 290 nm. PAL activity was expressed as milligrams of protein per minute (DA290 mg protein^− 1^ min^− 1^).

### Estimation of polyphenol oxidase activity

Polyphenol oxidase (PPO, EC 1.14.18.1) activity was measured by purpurogallin production [32]. Enzyme aliquots were incubated in 100 mM PPB (pH 6.0) with 100 mM catechol at 25 °C for 5 min. The reaction was stopped with 2.5 N H₂SO₄, and absorbance was measured at 495 nm. Polyphenol oxidase activity was expressed as milligrams of protein per minute (DA495 mg protein^− 1^ min^− 1^).

### Estimation of total phenolic content

Total phenolic content was quantified using the Folin-Ciocalteu method with gallic acid as the standard [33]. Shoots were extracted using 80% methanol at 80 °C for 90 min. The resulting extract was then centrifuged at 14,000 rpm for 15 min. Finally, the supernatant was mixed with 2 N Folin-Ciocalteu reagent and 20% Na_2_​CO_3_​. After 20 min of incubation at room temperature, the sample’s absorbance was measured at 725 nm using a spectrophotometer. We determined the phenolic content by comparing the sample’s absorbance to a gallic acid standard curve, expressing the results in mg g^− 1^ FW.

### Estimation of total flavonoid content

Total flavonoid content was measured using the method of Chang et al. [34], with quercetin as the standard. Methanol extracts were mixed with 10% aluminum nitrate and 1 M potassium acetate. After 40 min at room temperature, absorbance was measured at 415 nm. Flavonoid content was expressed as mg quercetin equivalents g⁻¹ FW.

### Estimation of phenolic and flavonoid profiles

Dried shoots were extracted with methanol at room temperature with stirring for 24 h, repeated three times. The extract was concentrated under reduced pressure using a rotary evaporator. The residue was suspended in 10% methanol and fractionated via liquid-liquid extraction with dichloromethane and ethyl acetate (three times each). Fractions were concentrated under reduced pressure to yield dichloromethane, ethyl acetate, and aqueous extracts.

### HPLC analysis

Phenolic compounds in the ethyl acetate extract were analyzed using an Agilent 1100 HPLC system with a UV/Vis detector and a C18 column (125 mm × 4.6 mm, 5 μm). The mobile phase consisted of solvent A (1% acetic acid in water) and solvent B (100% methanol). A gradient elution was used: 100% solvent A for 3 min, 50% solvent B for 5 min, increasing to 80% over 2 min, and to 100% over 5 min, maintained until 25 min, at a flow rate of 1 mL min⁻¹. The injection volume was 25 µg mL⁻¹. Phenolics were identified using Agilent ChemStation software and authentic standards.

**Table:** A: 1% acetic acid in water B: 100% methanol

Time (min)	% A	% B	Description
0.0	100	0	Initial: 100% A
3.0	100	0	Hold 100% A
8.0	50	50	50% B over 5 min
10.0	20	80	80% B over 2 min
15.0	0	100	100% B over 5 min
25.0	0	100	Hold 100% B
25.0 + (Stop)	–	–	End run

Flavonoids were analyzed on a C18 column (250 mm × 4.6 mm, 5 μm) using an isocratic mobile phase of 70% acetonitrile and 30% 1% formic acid in water, at 1 mL min⁻¹ for 25 min. Flavonoids were identified using authentic standards and Agilent ChemStation software.

**Table:** A: 1% formic acid in water. B: 100% acetonitrile

Time (min)	% A	% B	Flow Rate (mL min^− 1^)
0.0–25.0	30	70	1.0
25.0 (Stop)	-	-	End

### Estimation of metabolites

#### GC-MS analysis

Dichloromethane fractions were analyzed using a Shimadzu GC-MS-QP2010 system with an RTX-5MS column (30 m × 0.25 mm i.d. × 0.25 μm; Restek, USA). The oven temperature started at 70 °C, increasing at 40 °C min⁻¹ to 270 °C over 50 min. Helium was used as the carrier gas at 1.41 mL min⁻¹, with an injector temperature of 200 °C. Mass spectra were acquired at 70 eV, 60 mA filament emission, and 200 °C ion source temperature. Samples (1% v/w) were injected with a 1:15 split ratio, and full-scan data (m/z 50–500) were collected at 2.6 scans s⁻¹. Compounds were identified using the Wiley 275 L database (Thermo Fisher Scientific, USA) and confirmed by mass fragmentation patterns, with a similarity threshold of ≥ 70% for metabolite identification.

### Molecular docking

Molecular docking was performed using AutoDock Vina [35] to evaluate interactions between *A. vasica* metabolites (alkaloids, phenolics, and flavonoids) and the anti-ssDNA antigen-binding fragment (PDB: 1P7K). Ligand structures were retrieved from PubChem [36]. Protein and ligand preparation, cavity detection, and blind docking were conducted using the CB-Dock-2 server [18] with default parameters. Ten docking poses were generated, and the pose with the lowest Vina score (highest binding affinity) was selected for analysis.

### Statistical analysis

Experiments were conducted in duplicate, with four biological replicates per treatment (each consisting of three technical replicates), each comprising 25 jars. Data are presented as means ± standard deviation (SD). One-way ANOVA followed by Tukey’s post-hoc test was performed using SPSS 26.0 to identify significant differences (*P* ≤ 0.05). Pearson’s correlation coefficients were calculated to assess relationships between parameters under Co treatments. Principal component analysis (PCA) was used to analyze variations in the phenolic and flavonoid profiles.

## Results

### Leaf anatomy

**Fig. 1:**
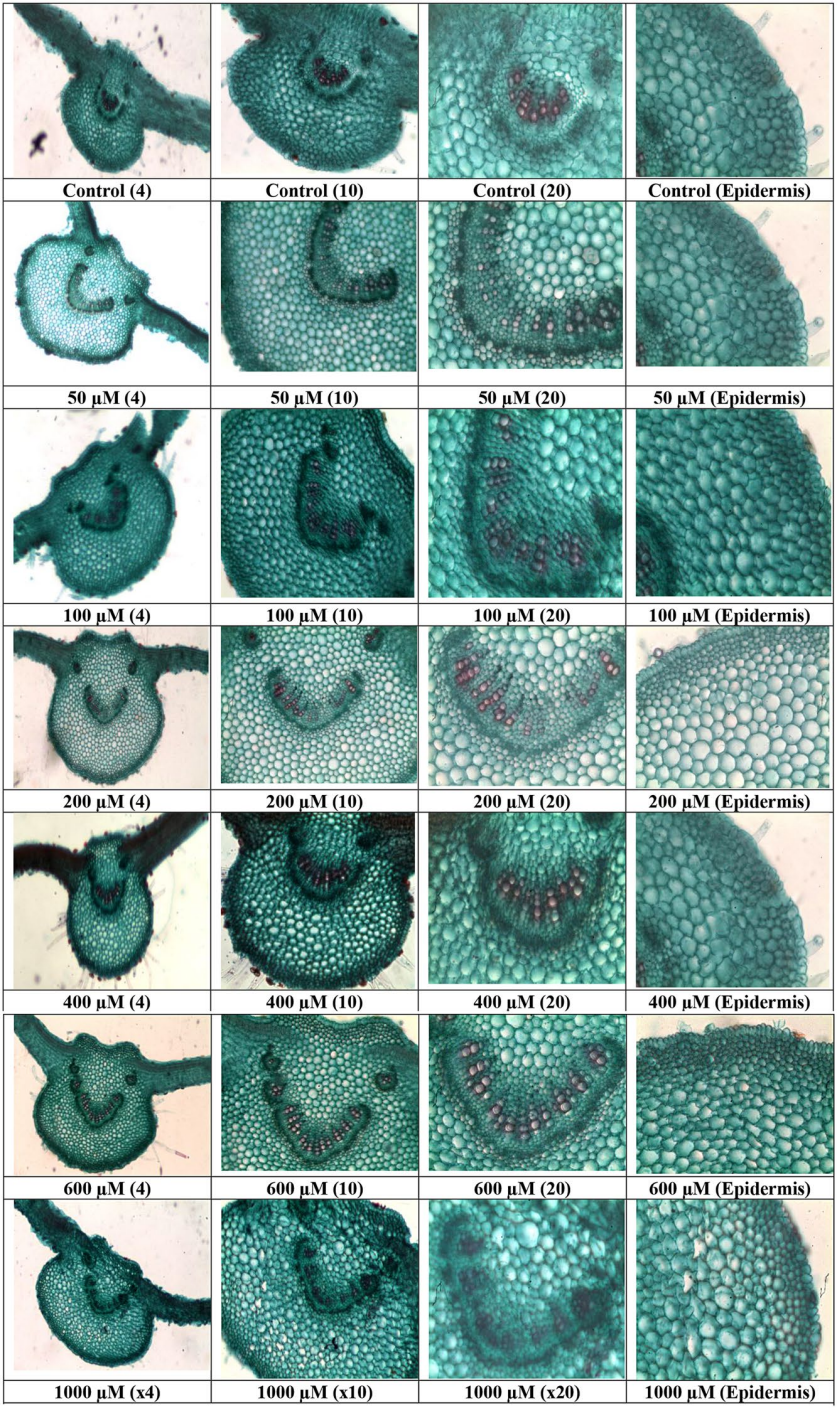
Leaf cross-section micrographs of *Adhatoda vasica* exposed to different concentrations of cobalt (0-1000 µM) for 30 days. (Obj x 4, 10 x, and 20 x)

Cobalt exposure altered *A. vasica* leaf anatomy, potentially impacting photosynthesis and stress resilience (Fig. 1). Transverse sections of the main vein showed a dose-dependent increase in xylem and phloem vessel counts with rising Co concentrations (50-1000 µM). Cell wall tortuosity also increased progressively, indicating structural adaptations to Co stress.

**Fig. 2:**
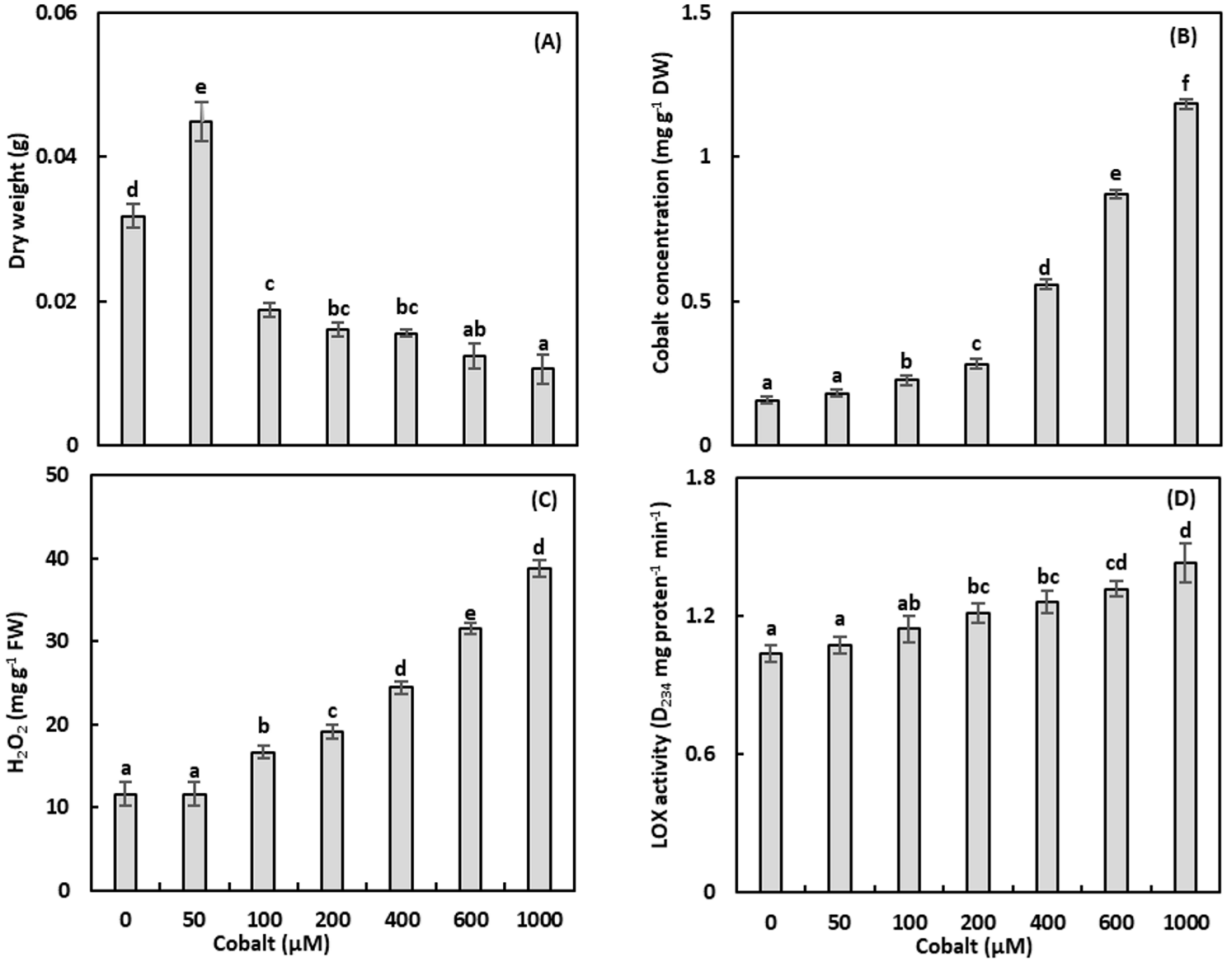
Dry weight (**A**), cobalt concentration (**B**), hydrogen peroxide (H_2_O_2_) levels (**C**), and the activity of lipoxygenase (LOX; **D**) of *Adhatoda vasica* proliferated shoots under the effect of different cobalt concentrations for 30 days. The data are means ± standard deviation (*n* = 4). The letters indicate statistically significant differences (*P* *≤* 0.05)

**Fig. 3:**
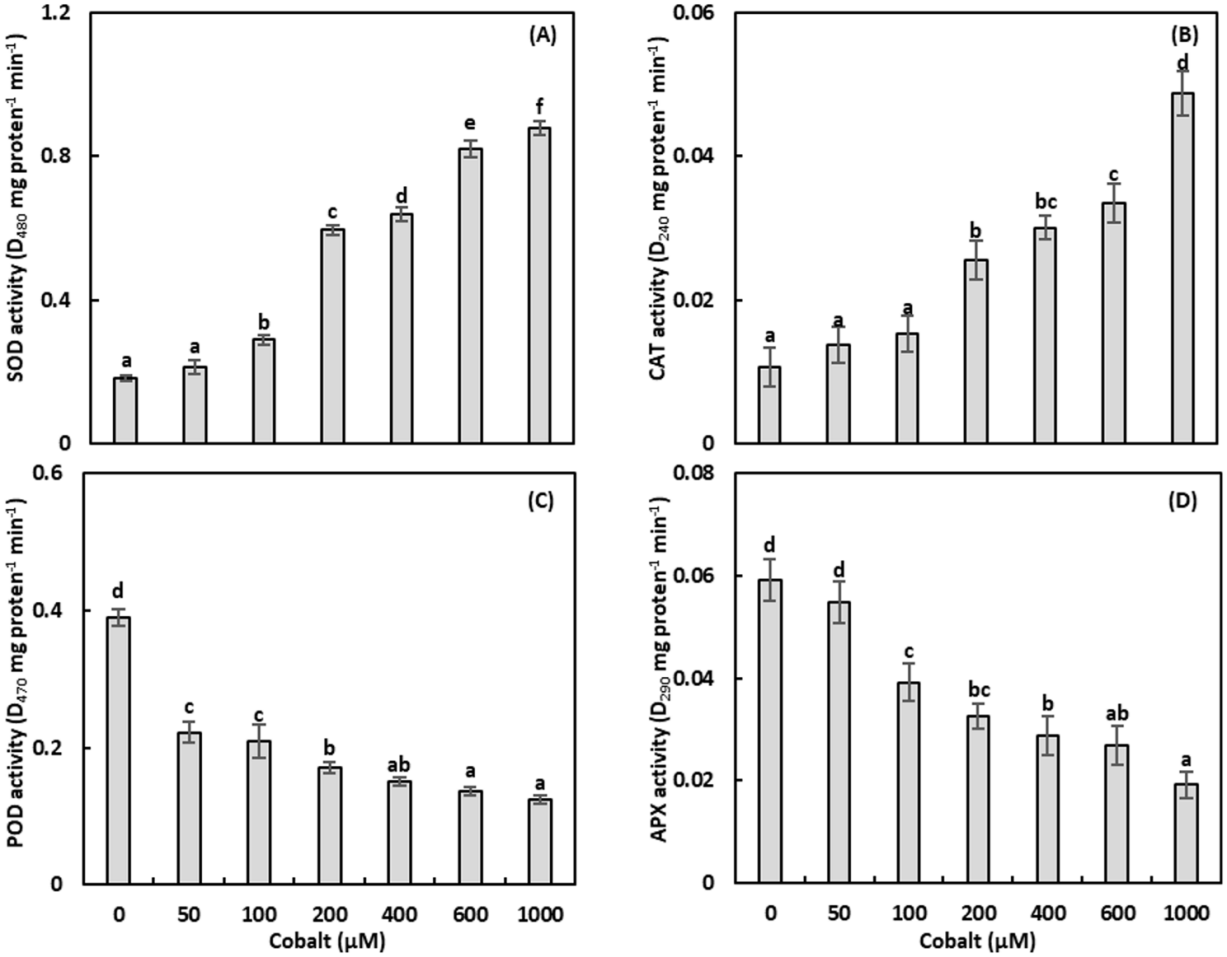
The activity of superoxide dismutase (SOD; **A**), catalase (CAT; **B**), peroxidase (POD; **C**), ascorbate peroxidase (APX; **D**) of *Adhatoda vasica* proliferated shoots under the effect of different cobalt concentrations for 30 days. The data are means ± standard deviation (*n* = 4). The letters indicate statistically significant differences (*P* *≤* 0.05)

**Fig. 4:**
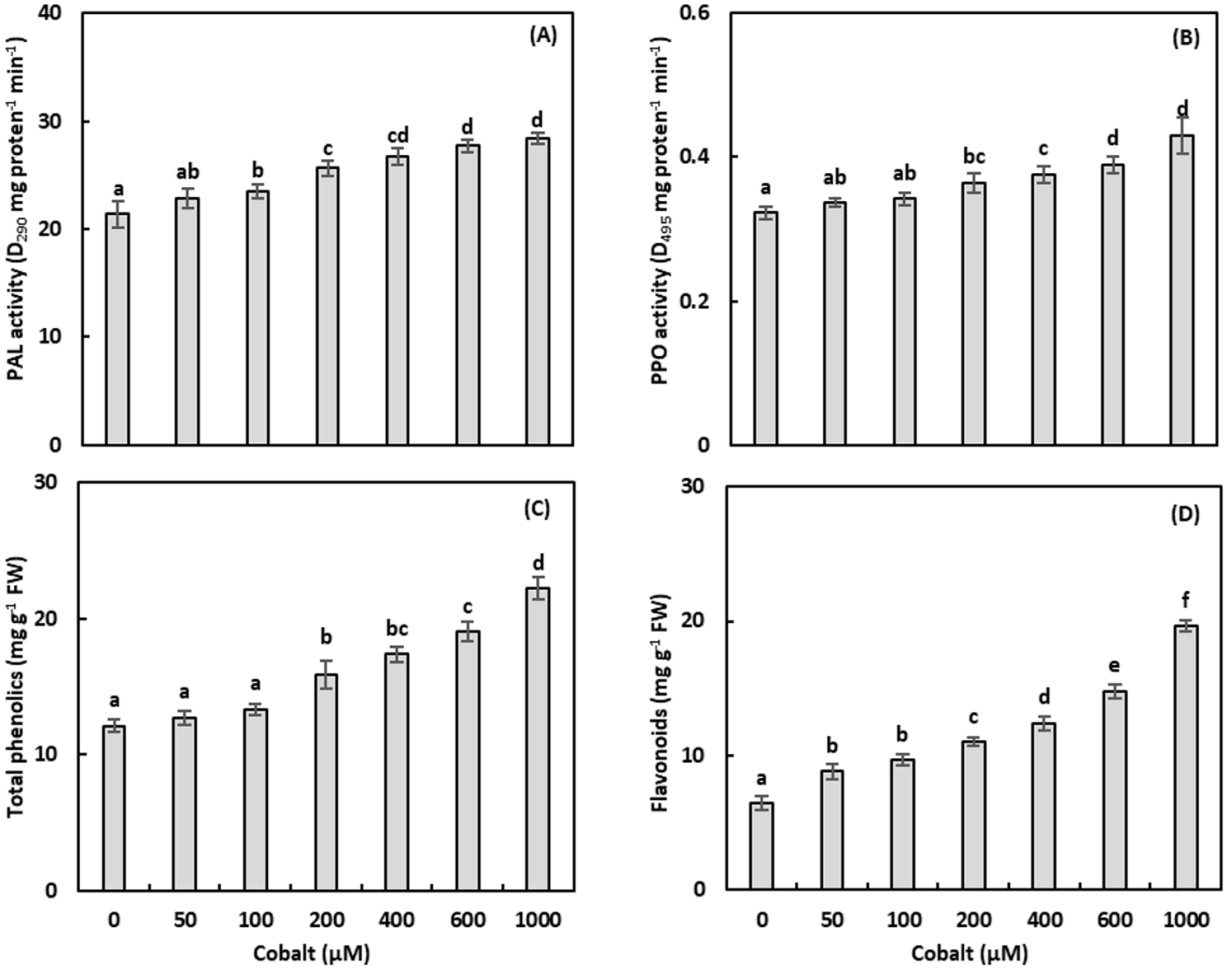
The activity of phenylalanine ammonia‑lyase (PAL; **A**), polyphenol oxidases (PPO; **B**), and the total phenolic (**A**), and total flavonoid concentrations of *Adhatoda vasica* proliferated shoots under the effect of different cobalt concentrations for 30 days. The data are means ± standard deviation (*n* = 4). The letters indicate statistically significant differences (*P* *≤* 0.05)

**Fig. 5:**
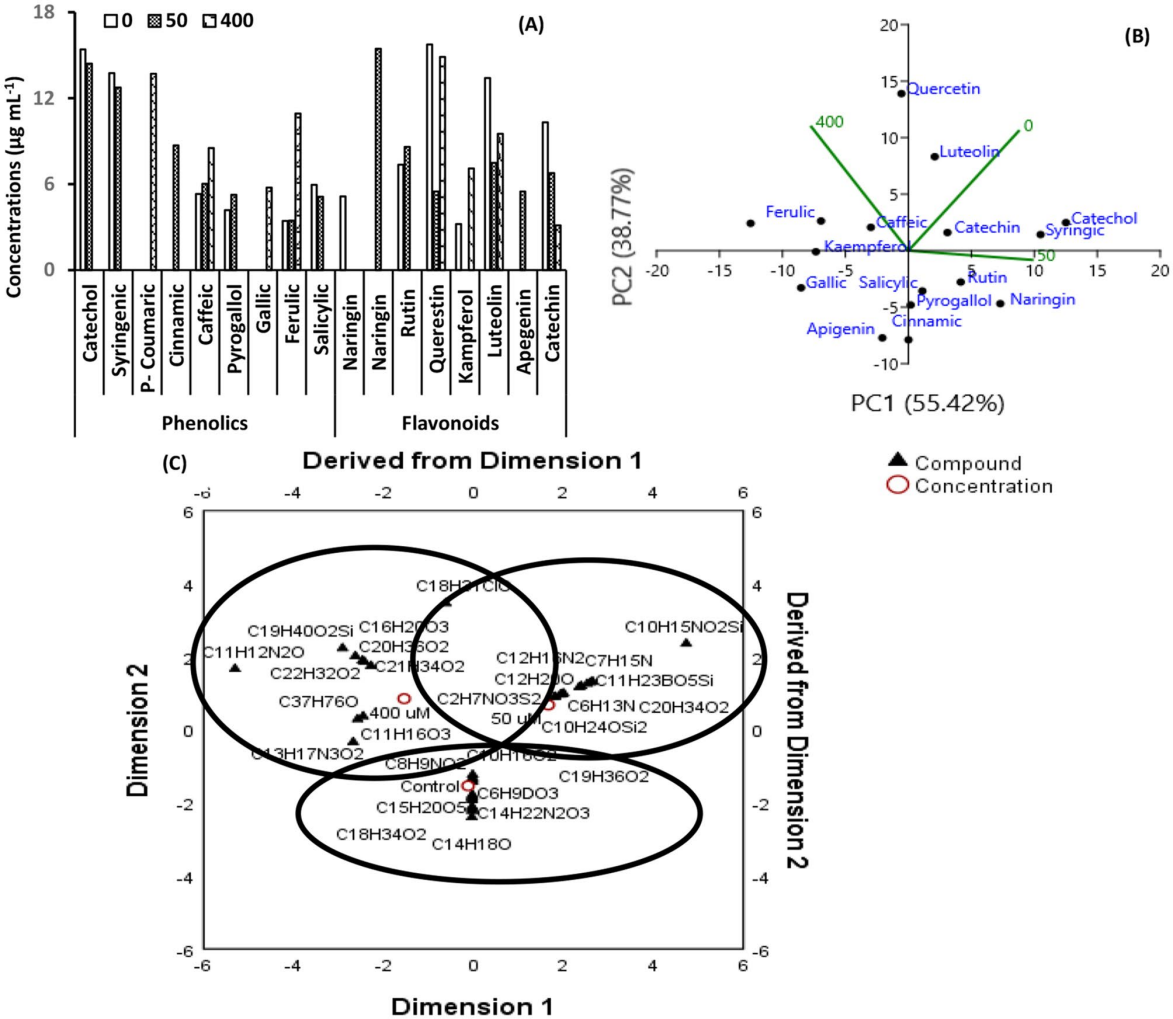
The concentration (**A**) and principal component analysis (PCA; **B**) of the phenolic, flavonoid, and gas chromatography/mass spectrometry (GC-MS; **C**) metabolic profiles of *Adhatoda vasica* proliferated shoots under the effect of different cobalt (0, 50, 400 µM) concentrations for 30 days

**Fig. 6:**
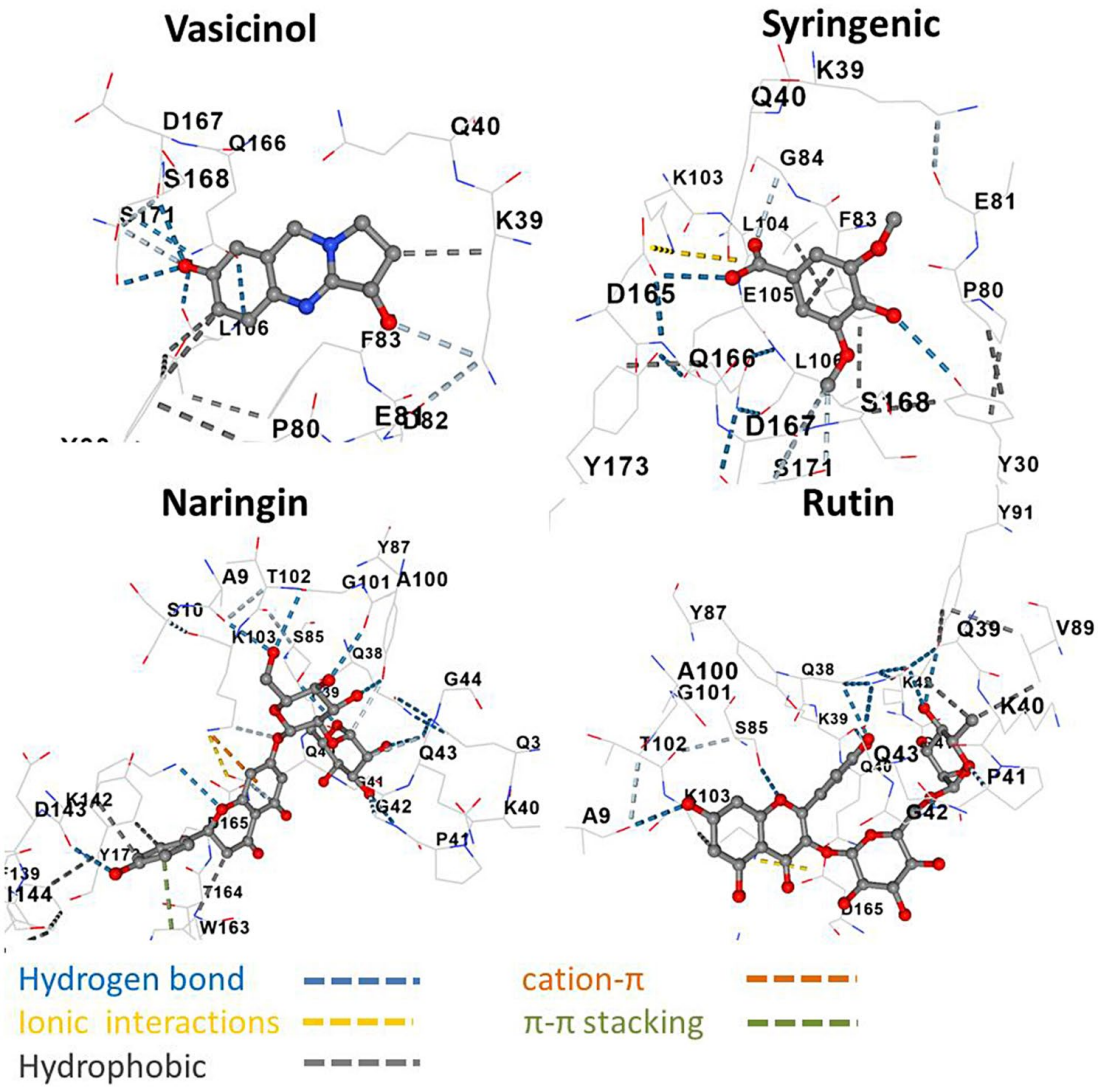
3D molecular interactions of vasicinol (Vina score − 7.0 kcal/mol), syringenic (-6.0), naringin (-9.2), and rutin (-8.7) with the active site of anti-ssDNA antigen-binding fragment (PDB code: 1P7K)

### Growth

Shoot growth was assessed by measuring the DW of proliferated *A. vasica* shoots under varying Co concentrations (Fig. 2A, Table S1). A statistically significant increase in shoot DW (*P* ≤ 0.05) was observed at 50 µM Co, showing a 41.45% gain over the control plants. However, higher Co concentrations (100–1000 µM) inhibited growth, with a maximum DW reduction of 66.86% at 1000 µM (*P* ≤ 0.05). A significant negative correlation (*r* = -0.677, *P* ≤ 0.01) was observed between DW and Co concentrations.

### Cobalt accumulation

Cobalt accumulation in *A. vasica* shoots was positively correlated with medium Co concentrations (*r* = 0.982, *P* ≤ 0.01; Fig. 2B, Table S1). At 50 µM Co, the shoot Co content increased 1.2-fold (non-significant) compared to the control plants. At 1000 µM Co, a significant 7.6-fold increase was observed (*P* ≤ 0.05), indicating enhanced Co uptake and storage at higher concentrations.

### Hydrogen peroxide

Hydrogen peroxide levels, a marker of oxidative stress, were quantified in *A. vasica* shoots (Fig. 2C, Table S1). At 50 µM Co, H₂O₂ levels remained unchanged. Higher Co concentrations (100–1000 µM) significantly increased H₂O₂ levels by 43.71-234.35% compared to the control (*P* ≤ 0.05), with a strong positive correlation (*r* = 0.980, *P* ≤ 0.01) between H₂O₂ and Co concentrations.

### Antioxidant enzyme activities

#### Lipoxygenase

Lipoxygenase activity, indicative of membrane lipid peroxidation, was measured in shoots (Fig. 2D, Table S1). Lipoxygenase activity was unaffected at 50–100 µM Co but increased significantly at 200–1000 µM, with a maximum increase of 38.16% at 1000 µM (*P* ≤ 0.05). A positive correlation (*r* = 0.899, *P* ≤ 0.01) was observed between LOX activity and Co concentrations.

### Superoxide dismutase

Superoxide dismutase activity, which mitigates superoxide radicals, was assessed (Fig. 3A, Table S1). Superoxide dismutase activity remained unchanged at 50 µM Co but increased significantly at 100–1000 µM, with a maximum enhancement of 385.22% at 1000 µM (*P* ≤ 0.05). A strong positive correlation (*r* = 0.904, *P* ≤ 0.01) was found between SOD activity and Co concentrations.

### Catalase

Catalase activity, responsible for H₂O₂ detoxification, was measured (Fig. 3B, Table S1). Catalase activity was unaffected at 50–100 µM Co but increased significantly at 200–1000 µM, with a maximum increase of 355.98% at 1000 µM (*P* ≤ 0.05). A strong positive correlation (*r* = 0.941, *P* ≤ 0.01) was observed between CAT activity and Co concentrations.

#### Peroxidase

Peroxidase activity, involved in H₂O₂-dependent oxidation, was evaluated (Fig. 3C, Table S1). Peroxidase activity decreased significantly with increasing Co concentrations, with a maximum reduction of 68.30% at 1000 µM (*P* ≤ 0.05). A negative correlation (*r* = -0.669, *P* ≤ 0.01) was found between POD activity and Co concentrations.

#### Ascorbate peroxidase

Ascorbate peroxidase activity, another H₂O₂-scavenging enzyme, was measured (Fig. 3D, Table S1). Ascorbate peroxidase activity was unaffected at 50 µM Co but decreased significantly at 100–1000 µM, with a maximum reduction of 67.55% at 1000 µM (*P* ≤ 0.05). A negative correlation (*r* = -0.809, *P* ≤ 0.01) was observed between APX activity and Co concentrations.

### Phenylalanine ammonia-lyase

Phenylalanine ammonia-lyase activity, linked to phenolic synthesis, was assessed (Fig. 4A, Table S1). Phenylalanine ammonia-lyase activity was unchanged at 50 µM Co but increased significantly at 100–1000 µM, with a maximum enhancement of 32.77% at 1000 µM (*P* ≤ 0.05). A positive correlation (*r* = 0.859, *P* ≤ 0.01) was found between PAL activity and Co concentrations.

### Polyphenol oxidase

Polyphenol oxidase activity, involved in phenolic oxidation, was measured (Fig. 4B, Table S1). Polyphenol oxidase activity was unchanged at 50–100 µM Co but increased significantly at 200–1000 µM, with enhancements of 12.80%, 16.44%, 20.58%, and 33.16% at 200, 400, 600, and 1000 µM, respectively (*P* ≤ 0.05). A strong positive correlation (*r* = 0.912, *P* ≤ 0.01) was observed between PPO activity and Co concentrations.

### Phenolic and flavonoid content

#### Total phenolic compounds

The total phenolic content in *A. vasica* shoots was quantified (Fig. 4C). Cobalt concentrations of 50–100 µM showed no significant effect, while 200–1000 µM significantly increased the phenolic content, with a maximum increase of 83.15% at 1000 µM (*P* ≤ 0.05). A strong positive correlation (*r* = 0.951, *P* ≤ 0.01) was observed between the phenolic content and cobalt concentrations.

### Total flavonoid compounds

Total flavonoid content, key to ROS scavenging, was measured (Fig. 4D). Cobalt concentrations of 50-1000 µM significantly increased flavonoid content, with a maximum increase of 204.29% at 1000 µM (*P* ≤ 0.05). A strong positive correlation (*r* = 0.962, *P* ≤ 0.01) was found between the flavonoid content and Co concentrations.

### Phenolic profiles

Phenolic profiles were analyzed via HPLC (Figs. 5 A-B, Table 1, Fig. S2). At 50 µM Co, catechol and syringic acid decreased by 6.43% and 7.27%, respectively, and were undetectable at 400 µM. The presence of p-coumaric acid (13.70 µg mL^− 1^) and gallic acid (5.76 µg mL^− 1^) was exclusive to the 400 µM Co group. Cinnamic acid (8.71 µg mL⁻¹) was detected only at 50 µM Co. Caffeic acid increased by 13.16% and 60.15% at 50 and 400 µM Co, respectively. Pyrogallol and salicylic acid showed biphasic responses, with pyrogallol increasing by 25.84% at 50 µM but undetectable at 400 µM, and salicylic acid decreasing by 13.95% at 50 µM and undetectable at 400 µM. Ferulic acid increased by 218.66% at 400 µM Co.

**Table 1:** HPLC analysis of *Adhatoda vasica* shoot Ethyl acetate extract: detected phenolics and flavonoids

	Com. No.	Compound Names	Retention Time (min)	Height (mAU)			Area (mAU.s)			Concentration µg/mL		
				0	50	400	0	50	400	0	50	400
**Phenolics**	1	**Catechol**	4	1300.102	1300.102	0	350.63	350.63	0	15.4	14.41	0
	2	**Syringic acid**	5	920.145	920.145	0	300.96	300.96	0	13.75	12.75	0
	3	**P-coumaric acid**	6	0	0	1300.05	0	0	450.12	0	0	13.7
	4	**Cinnamic acid**	7	0	605.076	0	0	270.42	0	0	8.71	0
	5	**Caffeic acid**	8	550.412	550.412	700.42	210.7	210.7	320.7	5.32	6.02	8.52
	6	**Pyrogallol**	9.4	350.117	350.117	0	100.05	100.05	0	4.18	5.26	0
	7	**Gallic acid**	10	0	0	490.32	0	0	201.33	0	0	5.76
	8	**Ferulic acid**	11	241.302	241.302	1020.41	95.26	95.26	410.32	3.43	3.45	10.93
	9	**Salicylic acid**	12	302.01	302.01	0	115.52	115.52	0	5.95	5.12	0
**Flavonoids**	1	**Naringin**	4.1	315.214	202.102	0	192.05	215.68	0	5.14	15.45	0
	2	**Rutin**	5.2	340.022	500.41	0	201.88	330.85	0	7.36	8.6	0
	3	**Quercetin**	7	1100.13	1201.011	1400.12	540.63	448.52	700.63	15.75	5.49	14.88
	4	**Kaempferol**	8	210.412	0	510.482	184.06	0	150.06	3.22	0	7.09
	5	**Luteolin**	9	1115.077	190.12	610.032	539.41	380.78	650.41	13.41	7.48	9.52
	6	**Apigenin**	10	0	177.03	0	0	189.58	0	0	5.49	0
	7	**Catechin**	12	560.411	195.255	180.451	420.7	320.09	99.41	10.31	6.78	3.12

**Table 2:** The profiles of GC-MS analysis compounds extracted and identified, based on a NIST library, from *Adhatoda vasica* proliferated shoots (30-day-old)-treated different Cobalt (0, 50, 400 µM) concentrations

No.	Compound Names	Molecular Formula	RT	Cobalt (µM)						Molecular Weight
			(min)	% Area under the peak			MF score			
				0	50	400	0	50	400	
1	6,8-Dioxabicyclo(3.2.1)octa N-3 L-OL-3-D1	C_6_H_9_DO_3_	5.06	0	1.17	0	0	766	0	131
2	6,8-Dioxabicyclo(3.2.1)octa N-3á-OL-2,2,4,4-D4	C6H_6_D_4_O_3_	5.14	0	0.24	0	0	775	0	134
3	2-(2-Aminoethylamino)ethanol	C_4_H_12_N_2_O	5.48	0.19	0	0	705	0	0	104.15
4	2-Decenoic Acid (monounsaturated medium-chain fatty acid)	C_10_H_18_O_2_	6.8	0.45	0	0	748	0	0	170.25
5	2-Nitrohept-2-en-1-ol	C_7_H_13_NO_3_	6.91	0.31	0	0	713	0	0	159.18
6	Benzoic Acid, TMS derivative	C_10_H_14_O_2_Si	8.67	0	0.84	0.66	0	701	877	194.3
7	(-)-Nepetalactol	C_10_H_16_O_2_	10	0	1.02	0	0	717	0	168.23
8	9,10-Secochola-5,7,10(19)-Trie ne-3,24-Diol, (3á,5Z,7E)-	C_24_H_38_O_2_	10.08	0	0	0.74	0	0	752	358.6
9	Ascaridole epoxide	C_10_H_16_O_3_	10.09	0	0.71	0	0	747	0	184.23
10	Methyl anthranilate	C_8_H_9_NO_2_	10.44	0	1.09	0	0	740	0	151.16
11	3-(1-Piperidinylmethyl)-1,3- Benzothiazole-2(3 H)-Thione #	C_13_H_16_N_2_S_2_	10.65	0	2.95	0	0	739	0	264.4
12	Piperidine, 2,3-dimethyl-	C_7_H_15_N	10.71	0	0	2.62	0	0	736	113.2
13	à-Pyrrolidone, 5-[3-hydroxybutyl]-	C_8_H_15_NO_2_	11.13	0	0	0.85	0	0	702	157.21
14	2-Methylpiperidine	C_6_H_13_N	11.14	0	2.2	0	0	808	0	99.17
15	Pyrrolizin-1,7-dione-6-carboxylic acid, methyl(ester)	C_9_H_11_NO_4_	12.35	0.4	0.8	0	704	708	0	197.19
16	{[4-({[4-(Diethyl amino)phenyl]methylidene}amino)benzoyl]amino}acetic acid	C_20_H_23_N_3_O_3_	13.95	0.88	0	0	702	0	0	454.4
17	Anthranilic acid, TMS derivative	C_10_H_15_NO_2_Si	13.96	0	2.92	2.35	0	933	940	209.32
18	Securinol B (Virosine B)	C_13_H_17_NO_3_	14.79	0.39	0	0	722	0	0	235.28
19	6,9-Octadecadiynoic acid, methyl ester	C_19_H_30_O_2_	15.34	0.62	0	0.37	707	0	761	290.4
20	11,14-Octadecadiynoic acid, methyl ester	C_19_H_30_O_2_	15.36	0	1.12	0	0	713	0	290.4
21	10-Methyltricyclo[4.3.1.1(2,5)]unde can-10-ol (10-Methyltricyclo[4.3.1.1(2,5)]undecan-10-ol)	C_12_H_20_O	15.6	0	2.23	0	0	752	0	180.29
22	3,7,11,14,18-Pentaoxa-2,19-disilaeic osane, 2,2,19,19-tetramethyl-	C_17_H_40_O_5_Si_2_	17.87	0	2.91	0	0	736	0	380
23	[1,1’-Bicyclopropyl]-2-octanoic acid,2’-hexyl-, methyl ester	C_21_H_38_O_2_	18.03	0.35	0	0.92	765	0	716	322.5
24	2(4 H)-benzofuranone, 5,6,7,7 A-tetrahydro-6-hydroxy-4,4,7 A-trimethyl-, (6 S-CIS)-	C_11_H_16_O_3_	19.46	0	3.4	1.38	0	845	825	196.24
25	9,12,15-Octadecatrienoic acid,2,3-bis(acetyloxy)propyl ester, (Z, Z,Z)-	C_25_H_40_O_6_	19.47	0.33	0	0	799	0	0	436.6
26	Acetamide, N-methyl-N-[4-[2-acetoxymethyl-1-p yrrolidyl]-2-butynyl]-	C_14_H_22_N_2_O_3_	20.39	0	1.74	0	0	731	0	266.34
27	Corymbolone (a eudesmane sesquiterpenoid)	C_15_H_24_O_2_	20.58	0	0.72	0	0	738	0	236.35
28	1,3,5-Triazine-2,4-diamine,6-chloro-n-ethyl-	C_5_H_8_ClN_5_	22.01	0.55	0.52	0	837	818	0	173.6
29	Ethanol, 2-(9-octadecenyloxy)-, (Z)-	C_20_H_40_O_2_	22.15	0.7	0	0	779	0	0	312.5
30	13-Heptadecyn-1-ol	C_17_H_32_O	22.18	0	0.89	0	0	738	0	252.4
31	Myristic acid, TMS derivative	C_17_H_36_O_2_Si	22.34	0.72	0	0	734	0	0	300.6
32	à-D-Galactopyranose, 6-O-(trimethylsilyl)-, cyclic 1,2:3,4-bis(methylboronate)	C_11_H_22_B_2_O_6_Si	22.35	0	0	0.64	0	0	708	300
33	Peganine (Vasicine)	C_11_H_12_N_2_O	22.84	1.97	3.94	3.08	724	819	766	188.23
34	7-Methyl-Z-tetradecen-1-ol acetate	C_17_H_32_O_2_	22.98	0.16	0	0	741	0	0	268.4
35	1 H-Cyclopenta(b)quinoline, 2,3,5,6,7,8-hexahydro-9-amino-	C_12_H_16_N_2_	23	0	2.67	0	0	751	0	188.27
36	1 H-Indole, 3-Methyl-2-(3-Methyl-3 H-IN DOL-3-YL)-	C_18_H_16_N_2_	23.13	0	0	11.14	0	0	882	260.3
37	5-Benzofuranacetic acid, 6-ethenyl- 2,4,5,6,7,7a-hexahydro-3,6-dimethyl-à-methylene-2-oxo-, methyl ester	C_16_H_20_O_4_	23.16	0.42	0	0	655	0	0	348.03
38	9-Amino-2,3,5,6,7,8-hexahydro-1 H-cyclopenta(b)quinoline	C_12_H_16_N_2_	23.16	0	3.5	0	0	748	0	188
39	Hexadecanoic acid, methyl ester	C_17_H_34_O_2_	23.63	4.44	0	5.99	821	798	0	270.5
40	n-Hexadecanoic acid	C_16_H_32_O_2_	24.5	6.13	0	0	842	0	0	256.42
41	Estra-1,3,5(10)-trien-17á-ol	C_18_H_24_O	24.6	0	2.03	0.8	0	711	742	256
42	1,5,9-Trimethyl-2-Oxatricyclo[7.3.0.0(3,8)]Dodec-3(8),4,6-Triene	C_14_H_18_O	24.86	0	12.42	1.98	0	884	852	202.29
43	Spiro[S-Indacene-2(1 H),2’-[2 H] Inden]-1-ONE, 1’,3,3’,5,6,7-Hexahydro-	C_20_H_18_O	25.98	0	4.5	0	0	837	0	274.4
44	Hexadecanoic acid, Trimethylsilyl Ester	C_19_H_40_O_2_Si	26.14	15.72	3.88	5.09	933	749	873	328.6
45	N-[5,9-Dimethyl-1-(3-Phenyl- 2-Oxiranyl)-4,8-Decadienyl Idene]-2-Phenyl-1-Aziridina Mine	C_28_H_34_N_2_O	26.41	0	0	0.34	0	0	708	414.6
46	Ethyl(9Z,12Z)-9,12-Octadecadienoate	C_20_H_36_O_2_	26.63	3.29	0	4.88	883	0	860	308.5
47	9-Octadecenoic acid (Z)-,Methyl Ester	C_19_H_36_O_2_	26.81	1.59	0	21.92	887	0	951	296.5
48	9-Octadecenoic acid (Z)-	C_18_H_34_O_2_	26.82	1.84	0.31	0	900	708	0	282.5
49	1 H-Cycloprop[e]azulen-7-ol, decahydro-1,1,7-trimethyl-4-methyle ne-, [1ar-(1aà,4aà,7á,7aá,7bà)]-	C_15_H_24_O	27.04	0	0	0.38	0	0	755	220.35
50	á-Vatirenene	C_15_H_22_	27.15	0	0.94	0	0	747	0	202.33
51	Octadecanoic acid, Methyl Ester	C_19_H_38_O_2_	27.37	0.93	0	0	799	0	0	298.5
52	Methyl-9,9,10,10 -Octadecanoate	C_19_H_38_O_2_	27.39	0	0.4	0	0	746	0	302
53	8,11,14-Eicosatrienoic acid, (Z, Z,Z)-	C_20_H_34_O_2_	27.53	2.82	0	0	823	0	0	306.5
54	9,12-Octadecadienoyl chloride, (Z, Z)-	C_18_H_31_ClO	27.62	3.97	0	2.63	836	0	787	298.9
55	5,8,11,14-Eicosatetraenoic acid, methyl ester, (all-Z)-	C_21_H_34_O_2_	27.7	0.88	3.04	0.7	785	789	754	318.5
56	Octadecanoic acid	C_18_H_36_O_4_	28.18	0	2	0	0	756	0	284
57	6-[1-(hydroxymethyl)vinyl]-4,8 A-dimethyl-1,2,4 A,5,6,7,8,8 A-octahydro-2-naphthalenol	C_15_H_24_O_2_	28.18	0	0	0.54	0	0	720	236
58	N-Isobutylundeca-2(E)-en-8,10-diyn amide	C_15_H_21_NO	28.28	0	0	0.32	0	0	749	231.33
59	Cinnamic acid, 4-hydroxy-3-methoxy-, (5-hydroxy-2-hydroxymethyl-6-[2-(4- hydroxy-3-methoxyphenyl)ethoxy]-4- (6-methyl-3,4,5-trihydroxytetrahydro pyran-2-	C_31_H_40_O_15_	28.79	0	0	0.19	709	0	0	652.6
60	9,12-Octadecadienoic acid (Z, Z)-, Trimethylsilyl Ester	C_21_H_40_O_2_Si	28.94	1.54	0	0	846	0	0	352.6
61	9,12,15-Octadecatrienoic acid, 2,3-bis[(trimethylsilyl)oxy]propyl ester, (Z, Z,Z)-	C_27_H_52_O_4_Si_2_	29.08	0.7	1.59	0.39	765	771	805	496.9
62	5,8,11-Eicosatrienoic acid, (Z)-, TMS derivative	C_23_H_42_O_2_Si	29.1	0.99	0.52	0.9	718	0	0	378
63	4 A,7-Ethano-4AH-Benzocyc Lohepten-5(2 H)-ONE, 1,3,4,6,7,8-Hexahydro-1,1,7-TR Imethyl-, (.+-.)-	C_16_H_24_O	29.57	0	0	0.4	0	0	886	232.36
64	Furoscrobiculin B	C_15_H_20_O_2_	29.62	0.42	6.03	0.97	771	785	758	232.32
65	2,2-dideutero octadecanal	C_18_H_36_O	30.15	1.55	0	0	741	0	0	270.5
66	Furosta-5,20(22)-dien-3,26-diol	C_27_H_42_O_3_	30.73	0.27	0.71	0.34	733	747	708	414.6
67	Cyclopropanedodecanoic acid,2-octyl-, methyl ester	C_24_H_46_O_2_	30.81	0.25	0	0	737	0	0	366.6
68	Ethyl iso-allocholate	C_26_H_44_O_5_	31.52	1.51	0.95	0.62	746	786	788	436.6
69	9-Hexadecenoic acid	C_16_H_30_O_2_	32.15	0.49	0	0	754	0	0	254.41
70	3 H-Cyclodeca[b]furan-2-one,4,9-dihydroxy-6-methyl-3,10-dimethy lene-3a,4,7,8,9,10,11,11a-octahydro-	C_15_H_20_O_4_	32.87	0.79	0.78	0.22	787	767	776	264.32
71	5-Benzofuranacetic acid, 6-ethenyl-2,4,5,6,7,7a-hexahydro-3,6 -dimethyl-à-methylene-2-oxo-, methyl ester	C_16_H_20_O_4_	32.9	0	0.72	0	0	814	0	276.33
72	4,7-Octadecadiynoic acid, methyl ester	C_19_H_30_O_2_	33.11	0.63	0	0	738	0	0	290.4
73	Hexanoic acid, pentadecyl ester	C_21_H_42_O_2_	33.4	0.89	0	0	742	0	0	326.6
74	7-Methyl-Z-tetradecen-1-ol acetate	C_17_H_32_O_2_	33.43	0.3	0.71	0	742	747	0	268
75	5,9-METHANO-5 H-[1,4,2,3]DIOX ADIAZOLO[2,3-A][1,2]DIAZEPIN =-2-AMINE	C_13_H_17_N_3_O_2_	33.82	1.96	3.52	0	777	777	0	247.29
76	Diisooctyl phthalate (Artifact)	C_24_H_38_O_4_	33.82	0	0	7.29	0	0	923	390.6
77	Pregan-20-one, 2-hydroxy-5,6-epoxy-15-methyl	C_22_H_34_O_3_	33.99	0	0.78	0.73	0	762	732	346
78	Androst-5,7-dien-3-ol-17-one	C_19_H_26_O_2_	34.01	0	1.47	0	0	749	0	286.4
79	Dodecanoic acid,2,3-bis(acetyloxy)propyl ester	C_19_H_34_O_6_	34.54	0.46	0	0	762	0	0	358.5
80	5á-Hydroxyneoverrucosa NE	C_20_H_34_O	35.36	0	0	1.78	0	0	745	290.5
81	Falcarinol	C_17_H_24_O	36.43	0.34	0	0	0	0	711	244.37
82	Cyclopropanebutanoic acid, 2-[[2-[[2-[(2-pentylcyclopropyl)meth yl]cyclopropyl]methyl]cyclopropyl] methyl]-, methyl ester	C_25_H_42_O_2_	36.96	0.3	0	1.16	773	0	755	374.6
83	Cholestan-3-ol, 2-methylene-,(3á,5à)-	C_28_H_48_O	38.22	0	0	0.5	0	0	819	400.7
84	Methyl 10,12-pentacosadiynoate	C_26_H_44_O_2_	41.58	0	0.41	0	0	804	0	388.6
85	Retinol, acetate	C_22_H_32_O_2_	44.26	3.24	0	0	785	0	0	328.5
86	1-Heptatriacotanol	C_37_H_76_O	44.26	3.24	1.24	0.77	788	782	778	537
87	Butyl 4,7,10,13,16,19-docosahexaenoate	C_26_H_40_O_2_	44.3	0	2.15	0	0	782	0	384.6
88	Lupeol	C_30_H_50_O_2_	46.91	27.45	0	0	822	0	0	426.7

**Table 3:** The Vina scores for the molecular docking of alkaloids, phenolics, and flavonoids with anti-ssDNA antigen-binding fragment (PDB code: 1P7K). Energies are in kcal/mol

Alkaloids	Score	Phenolics	Score	Flavonoids	Score
Pyridine-3-carbonitrile	-6.4	Catechol	-4.8	Naringin	-9.2
Peganine	-6.7	Syringic	-6.0	Rutin	-8.7
Furoscrobiculin B	-7.2	Caffeic	-6.4	Quercetin	-8.1
Anthranilic-silyl-ester	-6.2	Pyrogallol	-5.0	Kaempferol	-7.6
Eicosatetraenoic	-6.0	Ferulic	-6.4	Luteolin	-7.7
Octadecatrienoic	-6.1	Salicylic	-5.5	Catechin	-7.7
Octadecatrienoic-silyl-ester	-6.2	Cinnamic	-5.9	Apigenin	-7.6
Vasicine	-6.7	P-Coumaric	-6.2		
Vasicinone	-7.0				
Vasicinol	-7.0				
Vasicoline	-7.2				

### Flavonoid profiles

Flavonoid profiles were analyzed via HPLC (Figs. 5 A-B, Table 1, Fig. S2). Naringin and rutin showed biphasic responses, increasing by 200.58% and 16.85% at 50 µM Co, respectively, but were undetectable at 400 µM. Quercetin decreased by 65.14% at 50 µM and 5.52% at 400 µM. Kaempferol was undetectable at 50 µM but increased by 120.19% at 400 µM. Luteolin decreased by 44.22% at 50 µM and 29.01% at 400 µM. Apigenin (5.49 µg mL⁻¹) was detected only at 50 µM. Catechin decreased by 34.24% at 50 µM and 69.74% at 400 µM.

### Principal component analysis

Principal component analysis was used to assess variations in phenolic and flavonoid profiles (Fig. 5B). PC1 and PC2 explained 94.19% of the variance. Under control conditions, positive correlations were observed between catechol, syringic acid, catechin, luteolin, and quercetin. At 50 µM Co, correlations shifted, with negative correlations for these compounds and positive correlations for cinnamic acid, pyrogallol, naringin, and rutin. At 400 µM Co, negative correlations persisted for control and 50 µM parameters, with positive correlations for caffeic acid, ferulic acid, and kaempferol. Gallic acid, salicylic acid, and apigenin showed positive correlations across treatments.

### GC-MS analysis

GC-MS analysis identified 44, 42, and 36 metabolites in *A. vasica* shoots under 0, 50, and 400 µM Co treatments, respectively (Fig. 5C; Table 2, Table S2, Fig. S1). Component analysis showed clear separation between control and Co-treated samples. Nine metabolites, including vasicine (C₁₁H₁₂N₂O) and furoscrobiculin B (C₁₅H₂₀O₂), were consistent across treatments, with vasicine and furoscrobiculin B increasing by 100.00-1335.71% and 56.34-130.95% at 50 and 400 µM Co, respectively. Five metabolites, including hexadecanoic acid (C₁₉H₄₀O₂Si), decreased by 9.09–76.23% across Co treatments. Two metabolites (5,8,11,14-eicosatetraenoic acid and 9,12,15-octadecatrienoic acid) showed biphasic responses, increasing at 50 µM (245.45% and 127.14%) and decreasing at 400 µM (20.45% and 44.29%). Four metabolites were unique to 50 µM Co, with two increasing (e.g., pyrrolizin-1,7-dione-6-carboxylic acid, 100.00%) and two decreasing. Six metabolites were unique to 400 µM Co, with four increasing (e.g., 9-octadecenoic acid, 1278.62%) and two decreasing. Twenty-four metabolites were unique to the control, including methotrexate (C₂₀H₂₂N₈O₅) and lupeol (C₃₀H₅₀O₂).

### Molecular docking of some secondary metabolites

Molecular docking was performed to evaluate interactions between *A. vasica* metabolites (alkaloids, phenolics, and flavonoids) and the skin cancer-related protein anti-ssDNA antigen-binding fragment, a serine/threonine kinase involved in cytoskeletal regulation (Table 3). Phenolics showed the lowest binding affinities (-4.8 to -6.4 kcal/mol), with syringic acid forming hydrogen bonds with TYR 30, SER 171, ASP 167, and ASP 165, an ionic interaction with LYS 103, and a hydrophobic interaction with PHE 83 (Fig. 6). Flavonoids exhibited the highest affinities, with apigenin and kaempferol at -7.6 kcal/mol and naringin at -9.2 kcal/mol. Naringin formed 10 hydrogen bonds (with LYS 142, ASP 165, LYS 103, ALA 9, GLY 101, ALA 100, TYR 87, SER 85, GLN 43, GLY 42), a cation-π interaction with LYS 103, π-π stacking with TRP 163, and hydrophobic interactions with LYS 142, TYR 173, and TRP 163, indicating strong binding to the protein’s active site.

## Discussion

Cobalt stress influences the physiological and biochemical responses of *A. vasica*, revealing its potential as a hyperaccumulator and a source of therapeutic compounds. This study integrates anatomical, biochemical, and computational analyses to elucidate cobalt’s impact on shoot growth, antioxidant defenses, metabolite profiles, and protein interactions, addressing gaps in understanding Co tolerance in medicinal plants.

### Leaf anatomy and cobalt translocation

Heavy metal translocation from roots to shoots involves symplastic transport, xylem loading, and foliar uptake [37]. In *A. vasica*, Co exposure increased xylem and phloem vessel density in leaves in a dose-dependent manner, enhancing water and nutrient transport capacity (Fig. 1). This anatomical adaptation likely facilitates Co movement to shoots, as evidenced by a 7.6-fold increase in shoot Co content at 1000 µM (Fig. 2B). Similar adaptations in *Solanum nigrum* enable efficient translocation of Co, Cd, and Zn [38]. The increased cell wall tortuosity suggests structural reinforcement, potentially aiding Co sequestration. According to these results, *A. vasica* may be a Co hyperaccumulator with increased phloem mobility, which is a new finding for this species. Such traits position *A. vasica* as a candidate for phytoremediation, warranting further field-based studies to validate its efficacy.

### Growth responses

Cobalt’s function as a micronutrient supporting cellular homeostasis via interactions with Zn, Ni, and Fe [39] likely explains why the 50 µM treatment enhanced shoot dry weight by 41.45%. However, higher Co levels (100–1000 µM) reduced DW by up to 66.86%, reflecting toxicity-driven inhibition of cell division and photosynthesis (Fig. 2A) [22]. The negative correlation between DW and Co concentrations (*r* = -0.677, *P* ≤ 0.01) aligns with studies on wheat, where Co toxicity reduced growth [40]. These results highlight a biphasic response, with low Co promoting growth and high Co inducing stress.

### Oxidative stress and antioxidant responses

Cobalt stress disrupts electron transport in chloroplasts and mitochondria, increasing ROS production, including H₂O₂ [41]. Hydrogen peroxide levels rose by 43.71-234.35% at 100–1000 µM Co, correlating strongly with Co concentrations (*r* = 0.980, *P* ≤ 0.01; Fig. 2C). Elevated LOX activity at higher Co levels (up to 38.16% increase; Fig. 2D) indicates lipid peroxidation, contributing to membrane damage and growth inhibition (*r* = 0.899, *P* ≤ 0.01). Antioxidant enzymes responded variably: SOD and CAT activities increased significantly (up to 385.22% and 355.98%, respectively) at 200–1000 µM Co, reflecting robust ROS scavenging (Figs. 3A, B) [7]. Conversely, POD and APX activities decreased by up to 68.30% and 67.55%, respectively, suggesting that CAT is the primary H₂O₂ scavenger under Co stress (Figs. 3C, D). The negative correlations for POD (*r* = -0.669, *P* ≤ 0.01) and APX (*r* = -0.809, *P* ≤ 0.01) indicate species-specific responses, differing from Brassica napus, where POD and APX were upregulated [7]. Phenylalanine ammonia‑lyase and PPO activities increased at higher Co levels (up to 32.77% and 33.16%, respectively; Figs. 4A, B), enhancing phenolic and flavonoid synthesis to counter ROS (*r* = 0.859 and 0.912, *P* ≤ 0.01) [22].

### Secondary metabolite profiles

Cobalt stress altered secondary metabolite profiles in *A. vasica*, which are critical for stress tolerance and therapeutic potential. Total phenolic and flavonoid contents increased dose-dependently, with maximum increases of 83.15% and 204.29% at 1000 µM Co, respectively (Figs.4C, D). Specific phenolics showed varied responses: caffeic and ferulic acids increased significantly (up to 60.15% and 218.66%), while catechol, syringic acid, pyrogallol, and salicylic acid exhibited biphasic or reduced levels (Fig. 5A; Table 1). Flavonoids like naringin and rutin increased at 50 µM Co (200.58% and 16.85%) but were undetectable at 400 µM, suggesting stress-specific metabolic shifts (Table 1). Principal component analysis revealed that 94.19% of variance was explained by PC1 and PC2, with distinct correlation patterns under Co treatments (Fig. 5B). GC-MS analysis identified 44, 42, and 36 metabolites at 0, 50, and 400 µM Co, respectively, with vasicine and furoscrobiculin B increasing significantly (up to 1335.71% and 130.95%; Table 2). These shifts indicate Co-induced metabolic reprogramming, potentially enhancing antioxidant defenses and stress tolerance [42].

### Molecular docking and therapeutic potential

Molecular docking of *A. vasica* metabolites against the anti-ssDNA antigen-binding fragment (PDB code: 1P7K), a serine/threonine kinase linked to cancer cell invasion, revealed flavonoids as potent inhibitors. Naringin exhibited the highest binding affinity (-9.2 kcal/mol), forming 10 hydrogen bonds and multiple interactions (cation-π, π-π stacking, hydrophobic) with key residues (Fig. 6). Flavonoids outperformed phenolics (-4.8 to -6.4 kcal/mol) and alkaloids, with apigenin and kaempferol at -7.6 kcal/mol. Compared to 5-fluorouracil (-5.8 kcal/mol), a standard anti-cancer drug, naringin’s superior affinity suggests its potential as a skin cancer therapeutic [43]. These findings highlight that *A. vasica* metabolites, especially flavonoids, are promising therapeutic candidates that need to be validated in vitro.

### Implications and future directions

The study demonstrates that *A. vasica* tolerates Co by employing anatomical adaptations, upregulating antioxidants, and altering its metabolite profile. This suggests the plant could serve as a hyperaccumulator for phytoremediation. The finding of a biphasic growth response alongside dose-dependent metabolite changes highlights the delicate balance between cobalt’s role as both a beneficial micronutrient and a toxic agent. The high binding affinity of flavonoids like naringin to anti-ssDNA antigen-binding fragment suggests therapeutic applications, particularly for skin cancer. Future research should validate hyperaccumulation in field conditions, explore mechanistic links between anatomical changes and Co transport, and conduct in vitro assays to confirm docking predictions. These discoveries may improve the application of *A. vasica* in drug development and phytoremediation.

## Conclusion

This study elucidates the multifaceted responses of *A. vasica* shoots to Co stress, examining leaf anatomy, antioxidant enzyme activity, secondary metabolite profiles, and molecular interactions. Cobalt exposure induced dose-dependent anatomical changes, including increased xylem and phloem vessel density and cell wall tortuosity, which potentially enhanced Co translocation and supported the hyperaccumulator potential of *A. vasica*. Low Co concentrations (50 µM) promoted shoot DW by 41.45%, while higher concentrations (100–1000 µM) reduced DW by up to 66.86%, reflecting a biphasic growth response (*r* = -0.677, *P* ≤ 0.01). Shoot Co accumulation increased significantly, with a 7.6-fold rise at 1000 µM (*r* = 0.982, *P* ≤ 0.01), correlating with anatomical adaptations.

Oxidative stress markers, including H₂O₂ and LOX activity, increased dose-dependently (up to 234.35% and 38.16%, respectively), indicating ROS-mediated damage (*r* = 0.980 and 0.899, *P* ≤ 0.01). Antioxidant defenses were robust, with SOD and CAT activities rising significantly (up to 385.22% and 355.98%; *r* = 0.904 and 0.941, *P* ≤ 0.01), while POD and APX activities decreased (up to 68.30% and 67.55%; *r* = -0.669 and − 0.809, *P* ≤ 0.01), suggesting CAT’s primary role in H₂O₂ detoxification. Phenylalanine ammonia‑lyase and PPO activities increased (up to 32.77% and 33.16%; *r* = 0.859 and 0.912, *P* ≤ 0.01), driving phenolic and flavonoid synthesis.

Secondary metabolite profiles shifted significantly, with total phenolic and flavonoid contents increasing by 83.15% and 204.29% at 1000 µM Co, respectively. Specific metabolites exhibited biphasic responses: caffeic and ferulic acids increased (up to 60.15% and 218.66%), while naringin and rutin surged at 50 µM (200.58% and 16.85%) but were undetectable at 400 µM. Principal component analysis explained 94.19% of variance, revealing distinct metabolite correlations under Co stress. The number of metabolites identified by GC-MS decreased with increasing Co concentration (44 at 0 µM, 42 at 50 µM, and 36 at 400 µM). Furthermore, the dramatic increase in vasicine (1335.71%) and furoscrobiculin B (130.95%) suggests these compounds play a key role in cobalt tolerance.

Molecular docking against the skin cancer-related protein anti-ssDNA antigen-binding fragment (PDB code: 1P7K) showed flavonoids, particularly naringin (-9.2 kcal/mol), with superior binding affinities compared to phenolics (-4.8 to -6.4 kcal/mol) and 5-fluorouracil (-5.8 kcal/mol). Naringin’s interactions, including 10 hydrogen bonds, cation-π, π-π stacking, and hydrophobic contacts, highlight its potential as an anti-cancer agent for basal cell carcinoma. These findings underscore the Co tolerance mechanisms and therapeutic potential of *A. vasica*, paving the way for drug development and phytoremediation applications.

## Supplementary Information

Below is the link to the electronic supplementary material.

Supplementary Material 1

## Data Availability

Data will be made available on request.

## Abbreviations

AsA: Ascorbic acid
CAT: Catalase
Co: Cobalt
CoCl_2_: Cobalt chloride
DW: Dry weight
EDTA: Ethylenediaminetetraacetic acid
FW: Fresh weight
GC-MS: Gas chromatography-mass spectrometry
HPLC: High-performance liquid chromatography
H_2_O_2_: Hydrogen peroxide
IARC: International Agency for Research on Cancer
LOX: Lipoxygenase
MS: Murashige and Skoog medium
POD: Peroxidase
PAL: Phenylalanine ammonia-lyase
PPO: Polyphenol oxidase
PPB: Potassium phosphate buffer
ROS: Reactive oxygen species
PDB: Skin cancer-related proteins
SD: Standard deviation
SOD: Superoxide dismutase
TCA: Trichloroacetic acid
