# Supplementary material for: In vitro cobalt stress alters Adhatoda vasica anatomy, antioxidant defense, and metabolite profiles with docking insights
Source: BMC Biotechnol. 2025 Dec 27;26:12. doi: 10.1186/s12896-025-01088-9 (PMC12849156; doi:10.1186/s12896-025-01088-9)
Supplement: Supplementary file 1 — Supplementary Material 1 [file 12896_2025_1088_MOESM1_ESM.docx]

**Table S1** Pearson correlation analysis of *Adhatoda vasica* shoot parameters following 30-day of different cobalt concentrations exposure

|  | | | | | | | | | | | | | | | | | | | | | |
| --- | --- | --- | --- | --- | --- | --- | --- | --- | --- | --- | --- | --- | --- | --- | --- | --- | --- | --- | --- | --- | --- |
| **Parameter** | **Dry weight** | **Cobalt Concent.** | **H_2_O_2_** | **LOX** | **SOD** | | **CAT** | | **POD** | | **APX** | | **PAL** | | | **PPO** | | **Total**  **Phenolics** | | **Flavonoids** |  |
| **Dry weight** | 1 |  |  |  |  | |  | |  | |  | |  | | |  | |  | |  |  |
| **Cobalt concent.** | -.677^**^ | 1 |  |  |  | |  | |  | |  | |  | | |  | |  | |  |  |
| **H_2_O_2_** | -.778^**^ | 0.980^**^ | 1 |  |  | |  | |  | |  | |  | | |  | |  | |  |  |
| **LOX** | -.772^**^ | 0.899^**^ | 0.929^**^ | 1 |  | |  | |  | |  | |  | | |  | |  | |  |  |
| **SOD** | -.817^**^ | 0.904^**^ | 0.945^**^ | 0.915^**^ | 1 | |  | |  | |  | |  | | |  | |  | |  |  |
| **CAT** | -.735^**^ | 0.941^**^ | 0.953^**^ | 0.927^**^ | 0.940^**^ | | 1 | |  | |  | |  | | |  | |  | |  |  |
| **POD** | .625^**^ | -0.669^**^ | -0.730^**^ | -0.773^**^ | -0.803^**^ | | -0.764^**^ | | 1 | |  | |  | | |  | |  | |  |  |
| **APX** | .876^**^ | -0.809^**^ | -0.887^**^ | -0.879^**^ | -0.917^**^ | | -0.889^**^ | | 0.865^**^ | | 1 | |  | | |  | |  | |  |  |
| **PAL** | -.772^**^ | 0.859^**^ | 0.900^**^ | 0.899^**^ | 0.954^**^ | | 0.912^**^ | | -0.858^**^ | | -0.918^**^ | | 1 | | |  | |  | |  |  |
| **PPO** | -.706^**^ | 0.912^**^ | 0.927^**^ | 0.949^**^ | 0.896^**^ | | 0.942^**^ | | -0.747^**^ | | -0.875^**^ | | 0.906^**^ | | | 1 | |  | |  |  |
| **Total Phenolics** | -.742^**^ | 0.951^**^ | 0.967^**^ | 0.901^**^ | 0.952^**^ | | 0.964^**^ | | -0.760^**^ | | -0.896^**^ | | 0.911^**^ | | | 0.907^**^ | | 1 | |  |  |
| **Flavonoids** | -.705^**^ | 0.962^**^ | 0.971^**^ | 0.926^**^ | 0.918^**^ | | 0.964^**^ | | -0.789^**^ | | -0.876^**^ | | 0.893^**^ | | | 0.944^**^ | | 0.958^**^ | | 1 |  |
| **. Correlation is significant at the 0.01 level (2-tailed). | | | | | |  | |  | |  | |  | |  |  | |  | |  | | |

Cobalt concentration = Cobalt concent.

**Table S2** GC-MS data of the identified compounds in the *Adhatoda vasica* proliferated shoots dichloromethane extracts (30-day-old)-treated different cobalt (0, 50, 400 µM) concentrations.

| **No.** | **RT**  **(min)** | **Molecular Weight** | **Molecular Formula** | **MF score** | **MS/MS fragmentation** | **Identified compounds** |
| --- | --- | --- | --- | --- | --- | --- |
| 1 | 5.06 | 131 | C_6_H_9_DO_3_ | 766 | 131, 86, 58, 41 | 6,8-Dioxabicyclo(3.2.1)octa N-3L-OL-3-D1 |
| 2 | 5.14 | 134 | C6H_6_D_4_O_3_ | 755 | 134, 86, 58, 43 | 6,8-Dioxabicyclo(3.2.1)octa N-3á-OL-2,2,4,4-D4 |
| 3 | 5.48 | 104.15 | C_4_H_12_N_2_O | 705 | 104, 58, 42, 18 | 2-(2-Aminoethylamino)ethanol |
| 4 | 6.8 | 170.25 | C_10_H_18_O_2_ | 748 | 170, 84, 73, 55, 43, 29 | 2-Decenoic Acid (monounsaturated medium-chain fatty acid) |
| 5 | 6.91 | 159.18 | C_7_H_13_NO_3_ | 713 | 159, 95, 71, 55, 41 | 2-Nitrohept-2-en-1-ol |
| 6 | 8.67 | 194.3 | C_10_H_14_O_2_Si | 877 | 194, 179, 135, 105, 77, 51 | Benzoic Acid, TMS derivative |
| 7 | 10 | 168.23 | C_10_H_16_O_2_ | 717 | 168, 147, 135, 97, 71, 43 | (-)-Nepetalactol |
| 8 | 10.08 | 358.6 | C_24_H_38_O_2_ | 752 | 358, 145, 136, 118, 68, 59, 55, 44 | 9,10-Secochola-5,7,10(19)-Trie ne-3,24-Diol, (3á,5Z,7E)- |
| 9 | 10.09 | 184.23 | C_10_H_16_O_3_ | 747 | 184, 168, 150, 135, 125, 107, 69. 41 | Ascaridole epoxide |
| 10 | 10.44 | 151.16 | C_8_H_9_NO_2_ | 740 | 151, 119, 92, 65, 39 | Methyl anthranilate |
| 11 | 10.65 | 264.4 | C_13_H_16_N_2_S_2_ | 739 | 264, 167, 98 | 3-(1-Piperidinylmethyl)-1,3- Benzothiazole-2(3H)-Thione # |
| 12 | 10.71 | 113.2 | C_7_H_15_N | 736 | 113, 112, 98, 70, 56, 42, 28 | Piperidine, 2,3-dimethyl- |
| 13 | 11.13 | 157.21 | C_8_H_15_NO_2_ | 702 | 157, 129, 84, 56 | à-Pyrrolidone, 5-[3-hydroxybutyl]- |
| 14 | 11.14 | 99.17 | C_6_H_13_N | 802 | 99, 84, 56, 42 | 2-Methylpiperidine |
| 15 | 12.35 | 197.19 | C_9_H_11_NO_4_ | 708 | 197, 157, 113, 110, 98, 84, 55, 44 | Pyrrolizin-1,7-dione-6-carboxylic acid, methyl(ester) |
| 16 | 13.95 | 353 | C_20_H_23_N_3_O_3_ | 702 | 353, 338, 194, 120, 92, 65, 45 | {[4-({[4-(Diethyl amino)phenyl]methylidene}amino)benzoyl]amino}acetic acid |
| 17 | 13.96 | 209.32 | C_10_H_15_NO_2_Si | 933 | 209, 194, 176, 150, 120, 119, 92, 65 | Anthranilic acid, TMS derivative |
| 18 | 14.79 | 235.28 | C_13_H_17_NO_3_ | 722 | 235, 191, 140, 110, 55, 43 | Securinol B (Virosine B) |
| 19 | 15.34 | 290.4 | C_19_H_30_O_2_ | 707 | 290, 269, 205, 173, 145, 131, 105, 91, 79, 55, 41 | 6,9-Octadecadiynoic acid, methyl ester |
| 20 | 15.36 | 290.4 | C_19_H_30_O_2_ | 713 | 290, 259, 205, 173, 159, 145, 105, 91, 79, 55, 41 | 11,14-Octadecadiynoic acid, methyl ester |
| 21 | 15.6 | 180.29 | C_12_H_20_O | 752 | 180, 165, 137, 123, 95, 81, 67, 43 | 10-Methyltricyclo[4.3.1.1(2,5)]unde can-10-ol (10-Methyltricyclo[4.3.1.1(2,5)]undecan-10-ol) |
| 22 | 17.87 | 380 | C_17_H_40_O_5_Si_2_ | 701 | 380, 173, 161, 145, 131, 193, 73, 45 | 3,7,11,14,18-Pentaoxa-2,19-disilaeic  osane, 2,2,19,19-tetramethyl- |
| 23 | 18.03 | 322.5 | C_21_H_38_O_2_ | 765 | 322, 291, 237, 178, 165, 124, 109, 95, 81, 73, 55, 45 | [1,1'-Bicyclopropyl]-2-octanoic acid,2'-hexyl-, methyl ester |
| 24 | 19.46 | 196.24 | C_11_H_16_O_3_ | 845 | 196, 178, 163, 140, 135, 111, 57, 43 | 2(4H)-benzofuranone, 5,6,7,7A-tetrahydro-6-hydroxy-4,4,7A-trimethyl-, (6S-CIS)- |
| 25 | 19.47 | 436.6 | C_25_H_40_O_6_ | 799 | 436, 227, 213, 117, 97, 81, 55, 43 | 9,12,15-Octadecatrienoic acid,2,3-bis(acetyloxy)propyl ester,(Z,Z,Z)- |
| 26 | 20.39 | 266.34 | C_14_H_22_N_2_O_3_ | 731 | 266, 193, 124, 117, 82, 55, 43 | Acetamide, N-methyl-N-[4-[2-acetoxymethyl-1-p yrrolidyl]-2-butynyl]- |
| 27 | 20.58 | 236.35 | C_15_H_24_O_2_ | 738 | 236, 218, 203, 175, 153, 109, 93, 69, 55, 41 | Corymbolone (a eudesmane sesquiterpenoid) |
| 28 | 22.01 | 173.6 | C_5_H_8_ClN_5_ | 837 | 173, 158, 145, 111, 97, 85, 71, 55, 43 | 1,3,5-Triazine-2,4-diamine,6-chloro-n-ethyl- |
| 29 | 22.15 | 312.5 | C_20_H_40_O_2_ | 799 | 312, 250, 152, 124, 110, 96, 82, 69, 55, 41 | Ethanol, 2-(9-octadecenyloxy)-, (Z)- |
| 30 | 22.18 | 252.4 | C_17_H_32_O | 738 | 252, 219, 96, 82, 67, 55, 28 | 13-Heptadecyn-1-ol |
| 31 | 22.34 | 300.6 | C_17_H_36_O_2_Si | 734 | 300, 285, 145, 132, 117,73, 43 | Myristic acid, TMS derivative |
| 32 | 22.35 | 300 | C_11_H_22_B_2_O_6_Si | 708 | 300, 285, 183, 171, 159, 129, 117, 97, 84, 75, 44 | à-D-Galactopyranose, 6-O-(trimethylsilyl)-, cyclic 1,2:3,4-bis(methylboronate) |
| 33 | 22.84 | 188.23 | C_11_H_12_N_2_O | 766 | 188, 187, 159, 145, 131, 104, 77 | Peganine (Vasicine) |
| 34 | 22.98 | 268.4 | C_17_H_32_O_2_ | 741 | 268, 226, 171, 167, 143, 129, 111, 99, 85, 5543 | 7-Methyl-Z-tetradecen-1-ol acetate |
| 35 | 23 | 188.27 | C_12_H_16_N_2_ | 751 | 188, 187, 173, 160, 132, 77 | 1H-Cyclopenta(b)quinoline, 2,3,5,6,7,8-hexahydro-9-amino- |
| 36 | 23.13 | 260.3 | C_18_H_16_N_2_ | 882 | 260, 259, 245 | 1H-Indole, 3-Methyl-2-(3-Methyl-3H-Indol-3-YL)- |
| 37 | 23.16 | 276 | C_16_H_20_O_4_ | 655 | 276, 244, 176, 121, 91, 77, 53, 43 | 5-Benzofuranacetic acid, 6-ethenyl- 2,4,5,6,7,7a-hexahydro-3,6-dimethyl-à-methylene-2-oxo-,  methyl ester |
| 38 | 23.16 | 188 | C_12_H_16_N_2_ | 748 | 188, 187, 173, 160, 132, 104, 77 | 9-Amino-2,3,5,6,7,8-hexahydro-1H-cyclopenta(b)quinoline |
| 39 | 23.63 | 270.5 | C_17_H_34_O_2_ | 821 | 270, 239, 143, 129, 87, 74, 43 | Hexadecanoic acid, methyl ester |
| 40 | 24.5 | 256.42 | C_16_H_32_O_2_ | 842 | 256, 213, 129, 73, 60, 43 | n-Hexadecanoic acid |
| 41 | 24.6 | 256 | C_10_H_24_OSi_2_ | 711 | 256, 213, 185, 157, 129, 107, 97, 73, 57, 43 | Estra-1,3,5(10)-trien-17á-ol |
| 42 | 24.86 | 202.29 | C_14_H_18_O | 884 | 202, 188, 187 | 1,5,9-Trimethyl-2-Oxatricyclo[7.3.0.0(3,8)]Dodec-3(8),4,6-Triene |
| 43 | 25.98 | 274.4 | C_20_H_18_O | 699 | 274, 259, 257 | Spiro[S-Indacene-2(1H),2'-[2H] Inden]-1-ONE, 1',3,3',5,6,7-Hexahydro- |
| 44 | 26.14 | 328.6 | C_19_H_40_O_2_Si | 933 | 328, 313, 145, 132, 117, 75, 73, 43 | Hexadecanoic acid,Trimethylsilyl Ester |
| 45 | 26.41 | 414.6 | C_28_H_34_N_2_O | 708 | 414, 396, 282, 213, 131, 91, 69, 42 | N-[5,9-Dimethyl-1-(3-Phenyl- 2-Oxiranyl)-4,8-Decadienyl Idene]-2-Phenyl-1-Aziridina Mine |
| 46 | 26.63 | 308.5 | C_20_H_36_O_2_ | 883 | 308, 234, 220, 178, 150, 121, 109, 95, 81, 67, 55, 41 | Ethyl(9Z,12Z)-9,12-Octadecadieno ATE # |
| 47 | 26.81 | 296.5 | C_19_H_36_O_2_ | 887 | 296, 264, 163, 137, 112, 97, 83, 69, 55, 41 | 9-Octadecenoic acid (Z)-,methyl ester |
| 48 | 26.82 | 282.5 | C_18_H_34_O_2_ | 708 | 282, 265, 163, 137, 97, 83, 69, 55, 41 | 9-Octadecenoic acid (Z)- |
| 49 | 27.04 | 220.35 | C_15_H_24_O | 755 | 220, 205, 187, 159, 131, 119, 105, 91, 79, 55, 43, 41 | 1H-Cycloprop[e]azulen-7-ol, decahydro-1,1,7-trimethyl-4-methyle ne-, [1ar-(1aà,4aà,7á,7aá,7bà)]- |
| 50 | 27.15 | 202.33 | C_15_H_22_ | 747 | 202, 187, 159, 145, 131, 117, 105, 91, 77, 41 | á-Vatirenene |
| 51 | 27.37 | 298.5 | C_19_H_38_O_2_ | 799 | 298, 267, 255, 199, 143, 87, 74, 55, 43 | Octadecanoic acid, Methyl Ester |
| 52 | 27.39 | 302 | C_19_H_38_O_2_ | 746 | 302, 259, 143, 97, 87, 74, 57 | Methyl-9,9,10,10 -Octadecanoate |
| 53 | 27.53 | 306.5 | C_20_H_34_O_2_ | 823 | 306, 208, 150, 107, 95, 79, 67, 55, 41 | 8,11,14-Eicosatrienoic acid,(Z,Z,Z)- |
| 54 | 27.62 | 298.9 | C_18_H_31_ClO | 836 | 298, 264, 221, 165, 135, 123, 109, 95, 81, 67, 55, 41 | 9,12-Octadecadienoyl chloride,(Z,Z)- |
| 55 | 27.7 | 318.5 | C_21_H_34_O_2_ | 889 | 318, 247, 207, 150, 105, 93, 81, 79, 67, 41 | 5,8,11,14-Eicosatetraenoic acid, methyl ester, (all-Z)- |
| 56 | 28.18 | 284 | C_18_H_36_O_4_ | 756 | 284, 241, 199, 185, 129, 85, 73, 60, 55, 43, 41 | Octadecanoic acid |
| 57 | 28.18 | 236 | C_15_H_24_O_2_ | 720 | 236,218, 203, 187, 157, 143, 131, 119, 105, 91, 79 | 6-[1-(hydroxymethyl)vinyl]-4,8A-dimethyl-1,2,4A,5,6,7,8,8A-octahydro-2-naphthalenol |
| 58 | 28.28 | 231.33 | C_15_H_21_NO | 749 | 231, 202, 188, 174, 160, 131, 116, 91, 57, 41 | N-Isobutylundeca-2(E)-en-8,10-diyn amide |
| 59 | 28.79 | 652.6 | C_31_H_40_O_15_ | 709 | 652, 506, 330, 168, 151, 105, 55, 43 | Cinnamic acid, 4-hydroxy-3-methoxy-, (5-hydroxy-2-hydroxymethyl-6-[2-(4- hydroxy-3-methoxyphenyl)ethoxy]-4- (6-methyl-3,4,5-trihydroxytetrahydro pyran-2- |
| 60 | 28.94 | 352.6 | C_21_H_40_O_2_Si | 846 | 352, 337, 262, 129, 81, 75, 73, 67, 55, 41 | 9,12-Octadecadienoic acid (Z,Z)-, Trimethylsilyl Ester |
| 61 | 29.08 | 496.9 | C_27_H_52_O_4_Si_2_ | 744 | 496, 281, 221, 149, 133, 103, 73, 55, 41 | 9,12,15-Octadecatrienoic acid, 2,3-bis[(trimethylsilyl)oxy]propyl ester, (Z,Z,Z)- |
| 62 | 29.1 | 378 | C_23_H_42_O_2_Si | 718 | 378, 246, 192, 161, 129, 117, 79, 73, 67, 55, 41 | à-D-Mannopyranoside,Methyl, Cyclic 2,3:4,6-BIS(Butylboronate) |
| 63 | 29.57 | 232.36 | C_16_H_24_O | 886 | 232, 220, 217 | 4A,7-Ethano-4AH-Benzocyc Lohepten-5(2H)-ONE, 1,3,4,6,7,8-Hexahydro-1,1,7-TR Imethyl-, (.+-.)- |
| 64 | 29.62 | 232.32 | C_15_H_20_O_2_ | 758 | 232, 217, 189, 137, 105, 95, 51, 43, 41 | Furoscrobiculin B |
| 65 | 30.15 | 270.5 | C_18_H_36_O | 741 | 270, 250, 222, 152, 138, 124, 97, 83, 69, 57, 43 | 2,2-dideutero octadecanal |
| 66 | 30.73 | 414.6 | C_27_H_42_O_3_ | 733 | 414, 396, 357, 317, 215, 161, 153, 105, 93, 55, 43 | Furosta-5,20(22)-dien-3,26-diol |
| 67 | 30.81 | 366.6 | C_24_H_46_O_2_ | 737 | 366, 334, 266, 118, 83, 74, 69, 55, 41 | Cyclopropanedodecanoic acid,2-octyl-, methyl ester |
| 68 | 31.52 | 436.6 | C_26_H_44_O_5_ | 755 | 436, 400, 382, 272, 253, 129, 107, 81, 69, 55, 43, 41 | Ethyl iso-allocholate |
| 69 | 32.15 | 254.41 | C_16_H_30_O_2_ | 754 | 254, 236, 194, 138, 111, 97, 83, 69, 55, 41 | 9-Hexadecenoic acid |
| 70 | 32.87 | 264.32 | C_15_H_20_O_4_ | 787 | 264, 246, 228, 183, 163, 135, 91, 69, 55, 43, 41 | 3H-Cyclodeca[b]furan-2-one,4,9-dihydroxy-6-methyl-3,10-dimethy lene-3a,4,7,8,9,10,11,11a-octahydro- |
| 71 | 32.9 | 276.33 | C_16_H_20_O_4_ | 814 | 276, 244, 216, 176, 148, 121, 91, 77, 53, 43, 41 | 5-Benzofuranacetic acid, 6-ethenyl-2,4,5,6,7,7a-hexahydro-3,6 -dimethyl-à-methylene-2-oxo-, methyl ester |
| 72 | 33.11 | 290.4 | C_19_H_30_O_2_ | 738 | 290, 219, 178, 163, 131, 117, 105, 91, 55, 41 | 4,7-Octadecadiynoic acid, methyl ester |
| 73 | 33.4 | 326.6 | C_21_H_42_O_2_ | 742 | 326, 210, 217, 99, 55, 43, 41 | Hexanoic acid, pentadecyl ester |
| 74 | 33.43 | 268 | C_17_H_32_O_2_ | 747 | 268, 226, 213, 167, 129, 111, 99, 85, 43 | 7-Methyl-Z-tetradecen-1-ol acetate |
| 75 | 33.82 | 247.29 | C_13_H_17_N_3_O_2_ | 777 | 244, 177, 167, 149, 118, 91, 83, 70, 77, 55, 41 | 5,9-methano-5H-[1,4,2,3]Diox Adiazolo[2,3-A][1,2]Diazepin2-amine |
| 76 | 33.82 | 390.6 | C_24_H_38_O_4_ | 923 | 390, 279, 167, 149, 70,57 | Diisooctyl phthalate (Artifact) |
| 77 | 33.99 | 346 | C_22_H_34_O_3_ | 762 | 346, 328, 302, 211, 122, 91, 67, 55 | Pregan-20-one, 2-hydroxy-5,6-epoxy-15-methyl |
| 78 | 34.01 | 286.4 | C_19_H_26_O_2_ | 749 | 286, 253, 227, 143, 105, 91, 69, 55 | Androst-5,7-dien-3-ol-17-one |
| 79 | 34.54 | 358.5 | C_19_H_34_O_6_ | 762 | 358, 218, 183, 159, 129, 73, 45 | Dodecanoic acid,2,3-bis(acetyloxy)propyl ester |
| 80 | 35.36 | 290.5 | C_20_H_34_O | 745 | 290, 275, 248, 191, 163, 149, 123, 95, 69, 55, 41 | 5á-Hydroxyneoverrucosa NE |
| 81 | 36.43 | 244.37 | C_17_H_24_O | 711 | 244, 229, 217, 187, 173, 159, 117, 91, 77, 55 | Falcarinol |
| 82 | 36.96 | 374.6 | C_25_H_42_O_2_ | 773 | 374, 334, 298, 270, 227, 175, 149, 135, 121, 95, 87, 81, 74, 55 | Cyclopropanebutanoic acid, 2-[[2-[[2-[(2-pentylcyclopropyl)meth yl]cyclopropyl]methyl]cyclopropyl] methyl]-, methyl ester |
| 83 | 38.22 | 400.7 | C_28_H_48_O | 819 | 400, 382, 367, 272, 253, 129, 107, 83, 81, 69, 55, 43, 41 | Cholestan-3-ol, 2-methylene-,(3á,5à)- |
| 84 | 41.58 | 388.6 | C_26_H_44_O_2_ | 804 | 388, 357, 248, 159, 147, 133, 119, 91, 79, 55, 41 | Methyl 10,12-pentacosadiynoate |
| 85 | 44.26 | 328.5 | C_22_H_32_O_2_ | 785 | 328, 268, 211, 183, 159, 157, 145, 119, 105, 91, 55, 43 | Retinol, acetate |
| 86 | 44.26 | 537 | C_37_H_76_O | 788 | 537, 273, 257, 229, 190, 147, 107, 95, 81, 69, 55, 43 | 1-Heptatriacotanol |
| 87 | 44.3 | 384.6 | C_26_H_40_O_2_ | 782 | 384, 355, 327, 311, 248, 215, 199, 159, 133, 105, 91, 79, 67 | Butyl 4,7,10,13,16,19-docosahexaenoate |
| 88 | 46.91 | 426.7 | C_30_H_50_O_2_ | 822 | 426, 411, 315, 234, 218, 207, 147, 121, 109, 95, 81, 68, 43 | Lupeol |


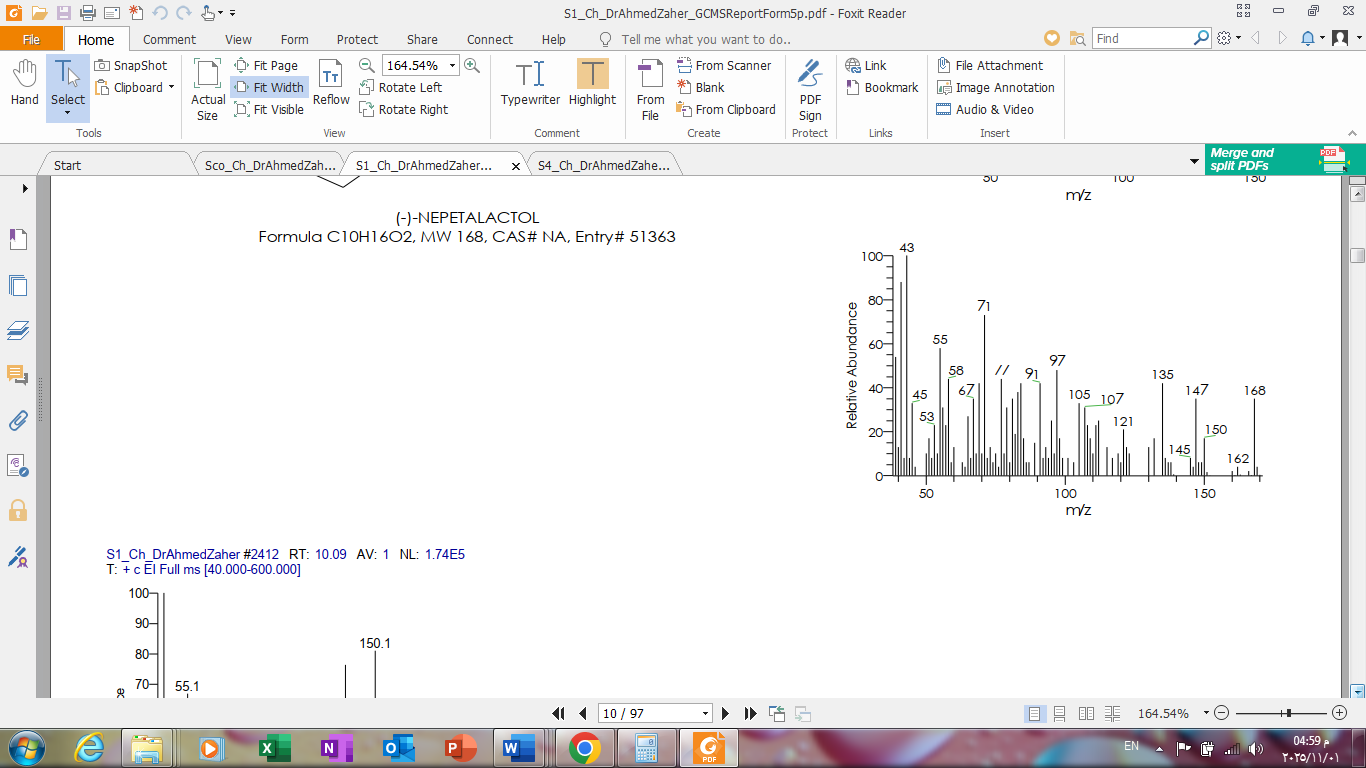


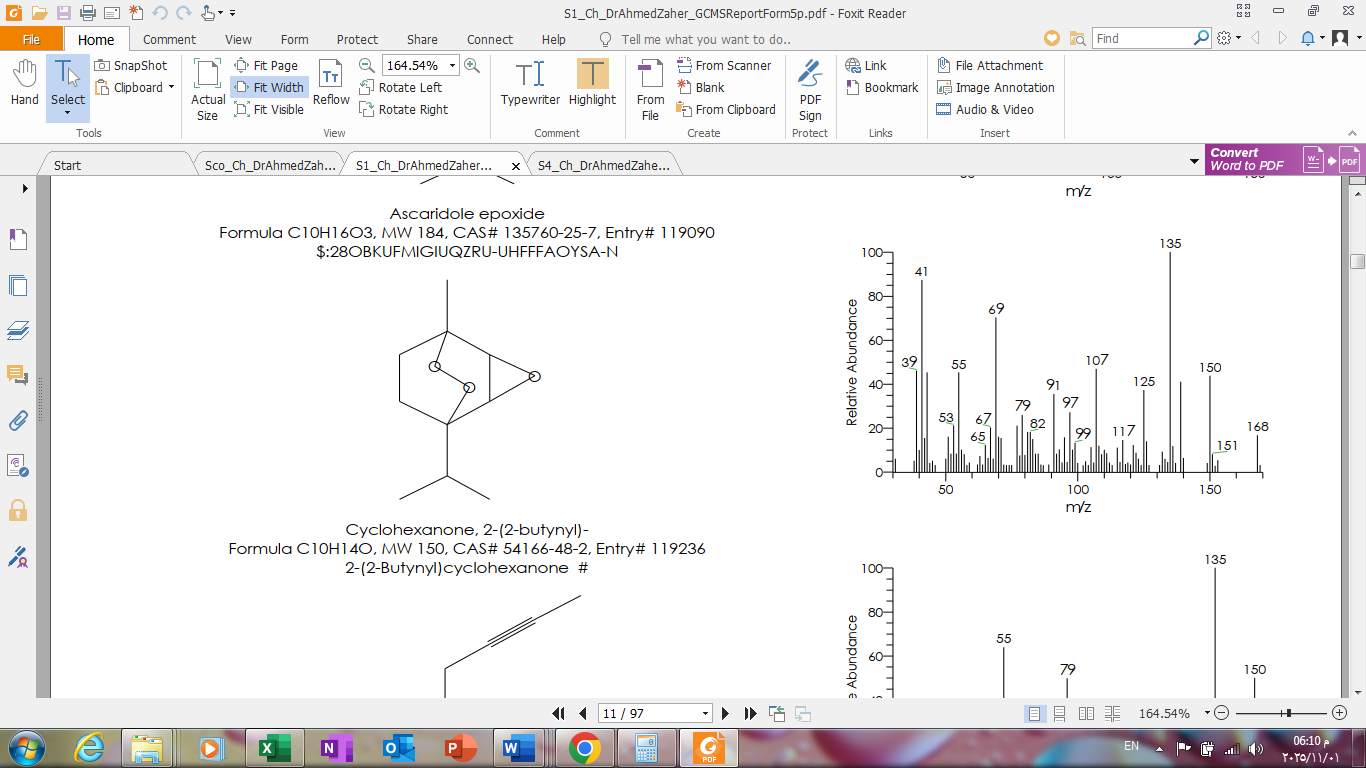


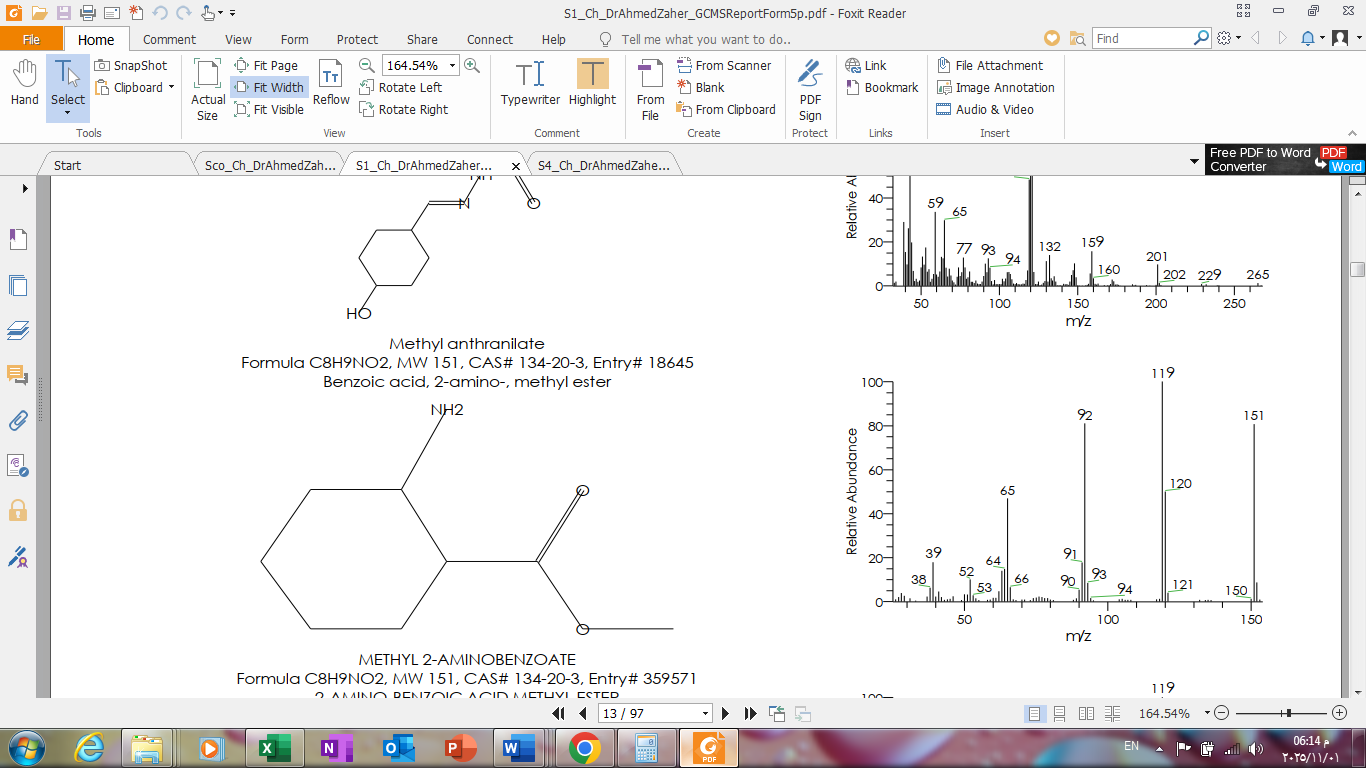


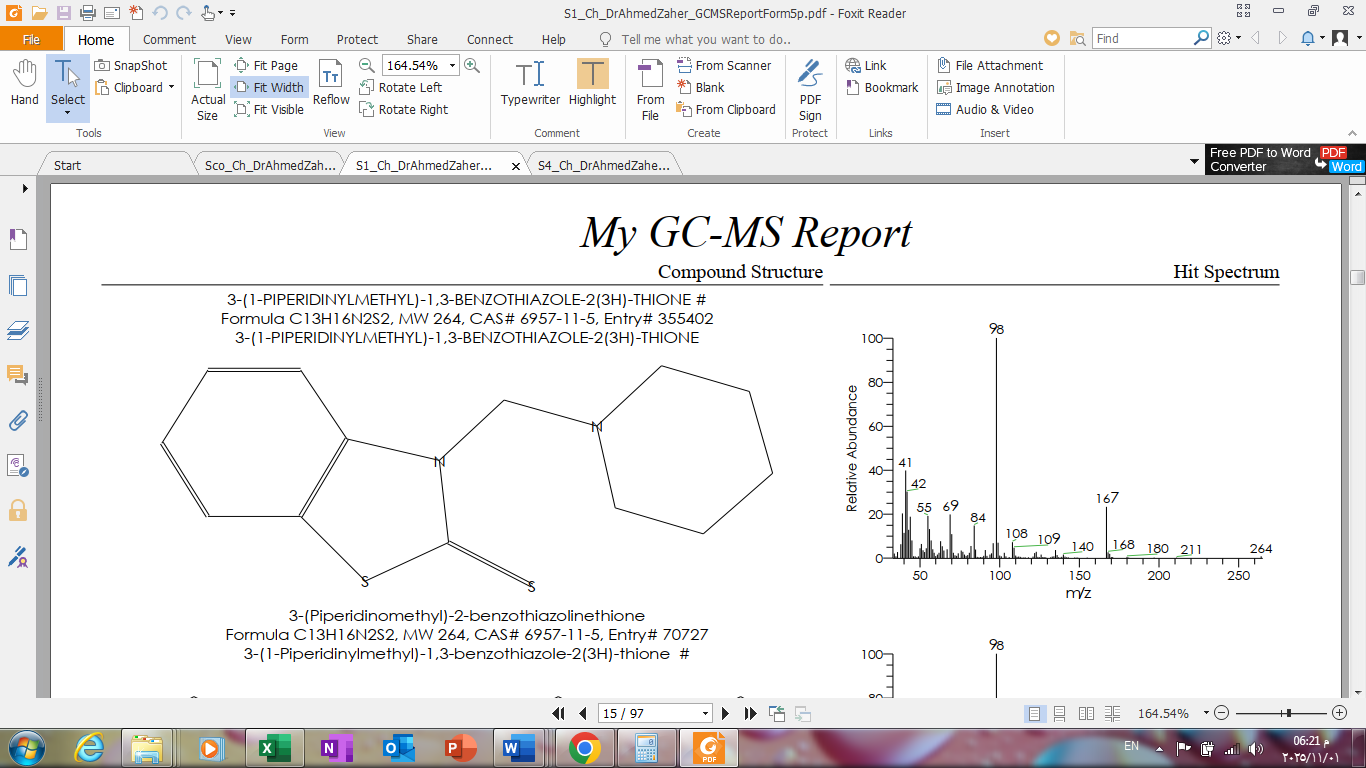


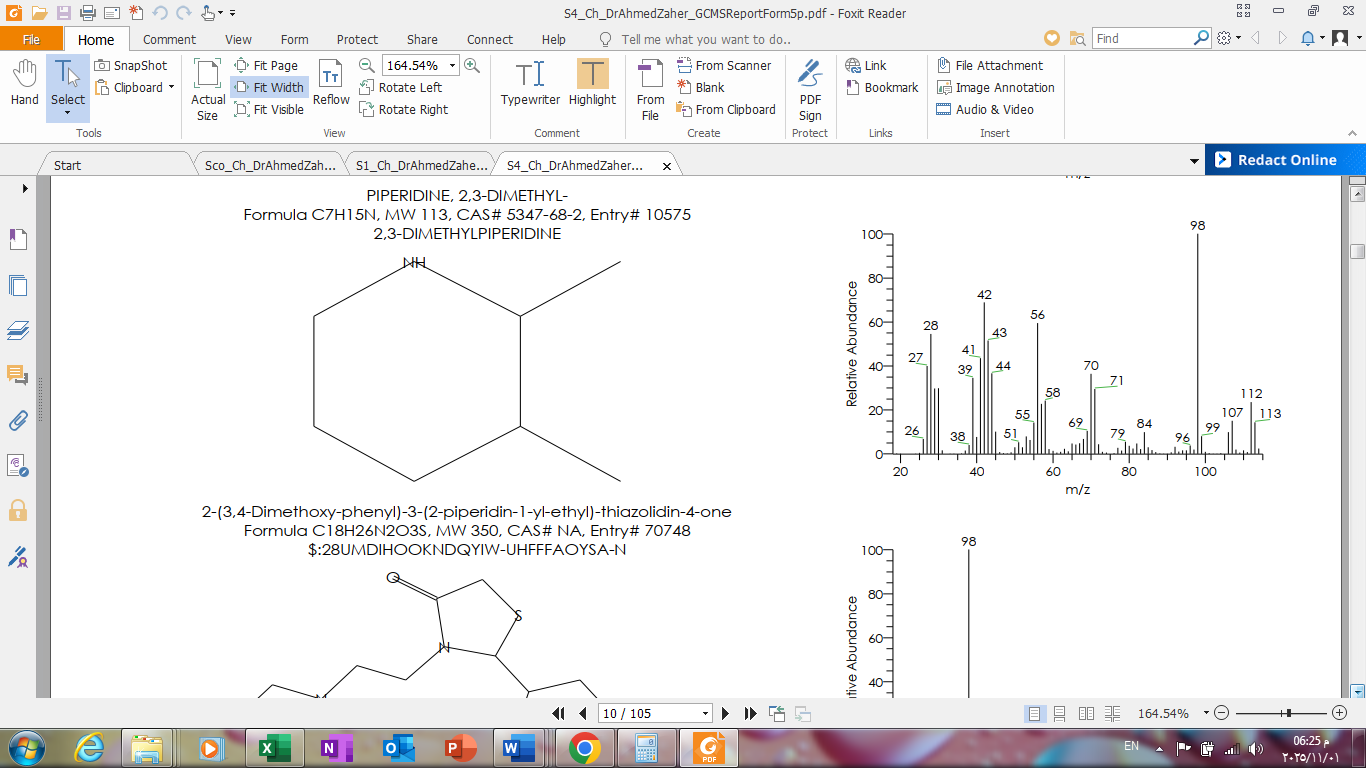


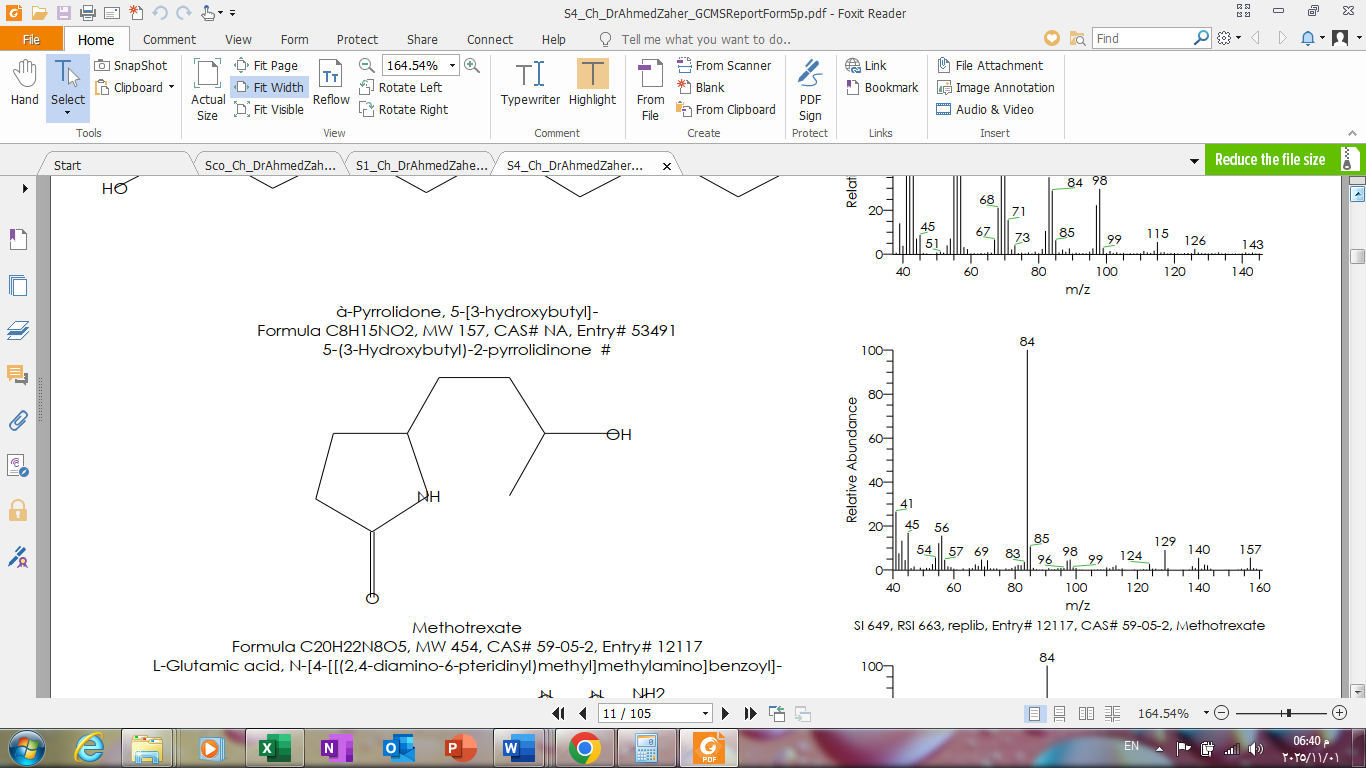


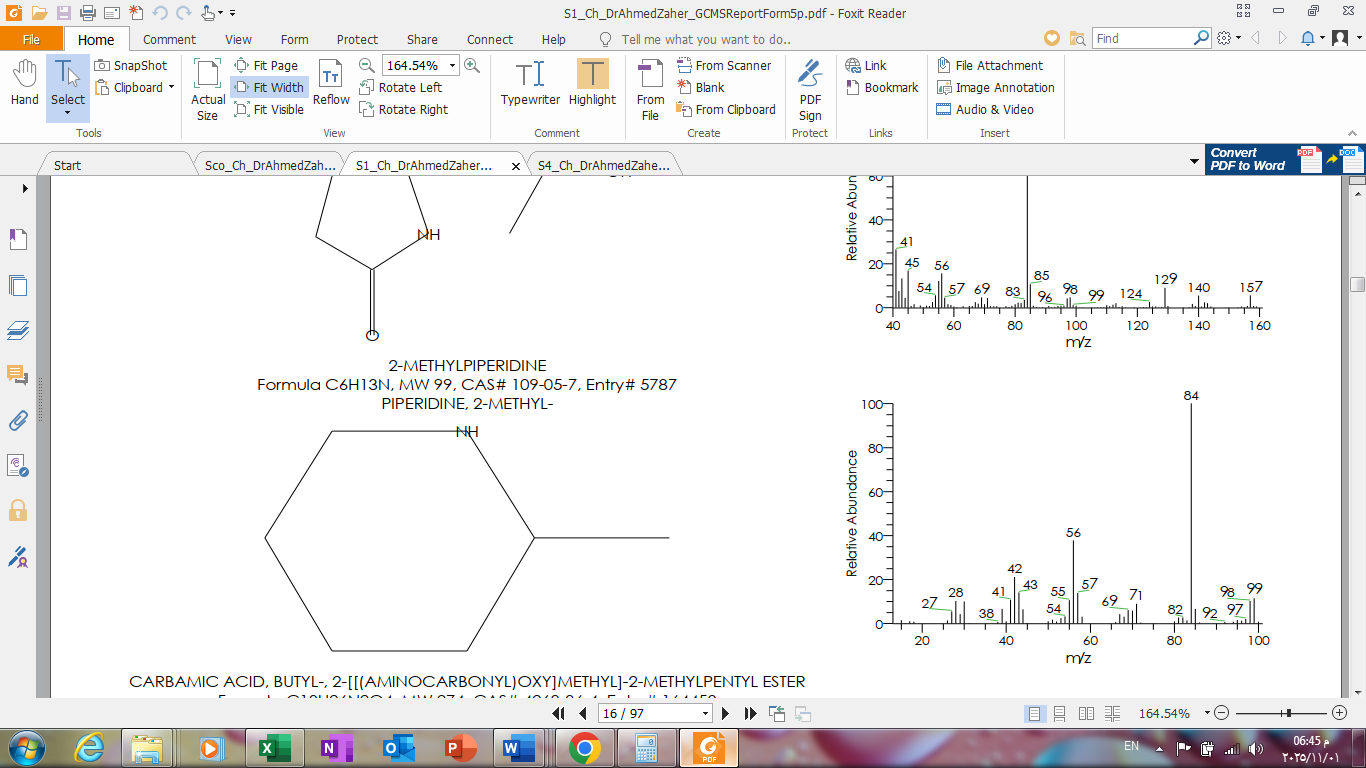


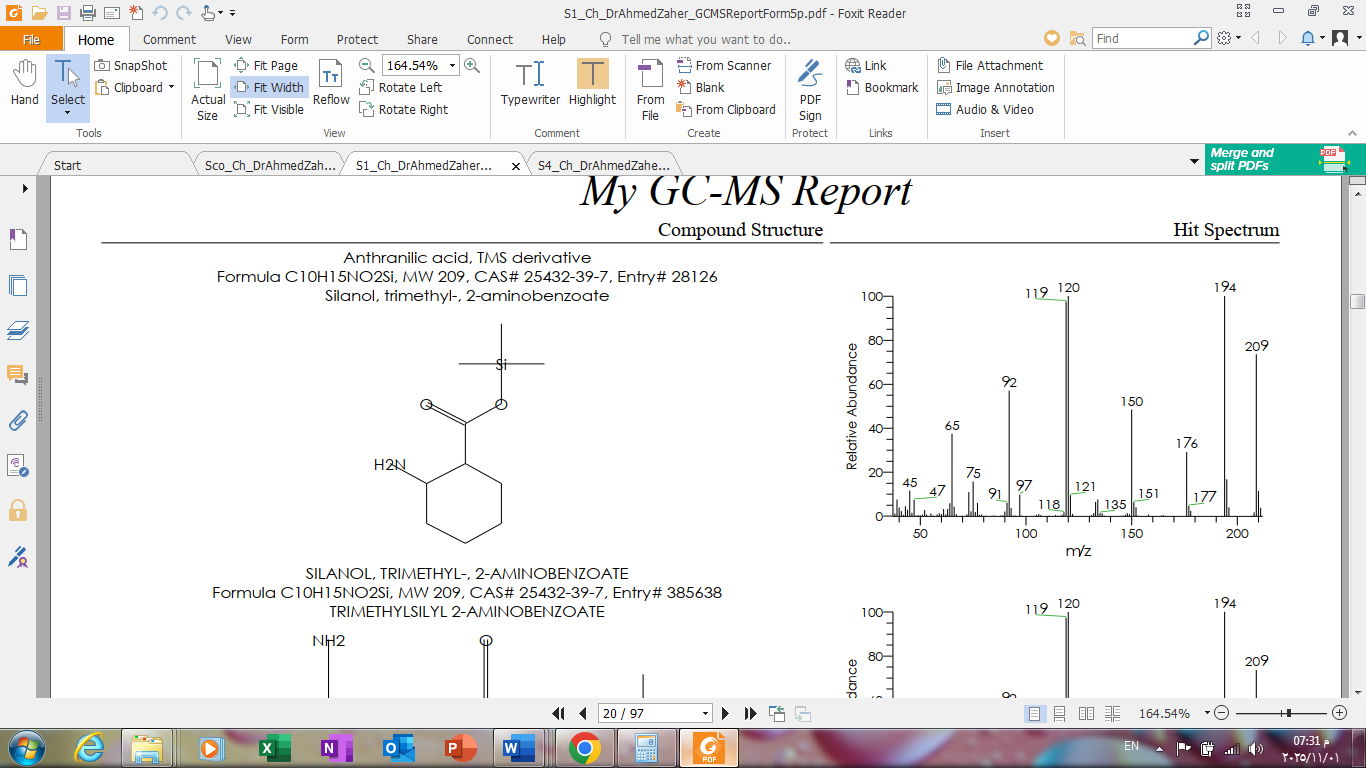


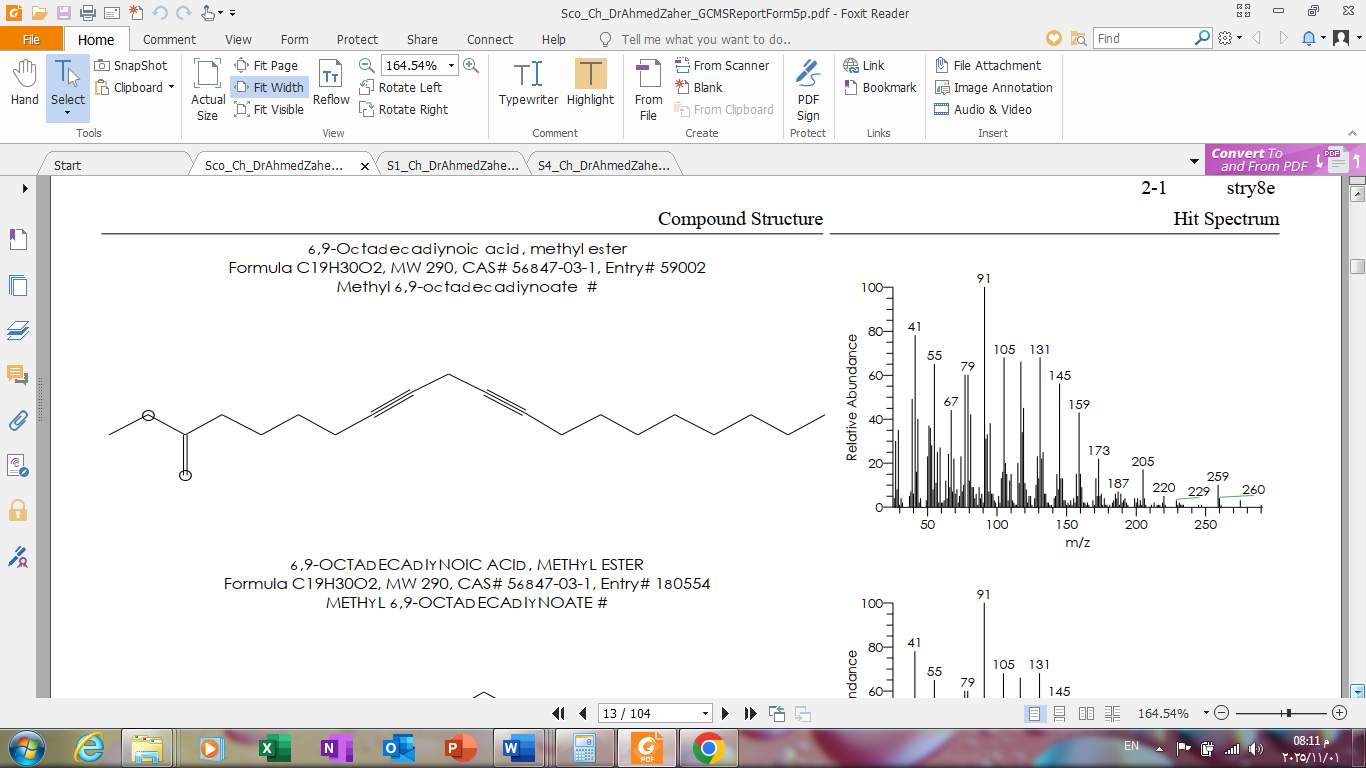


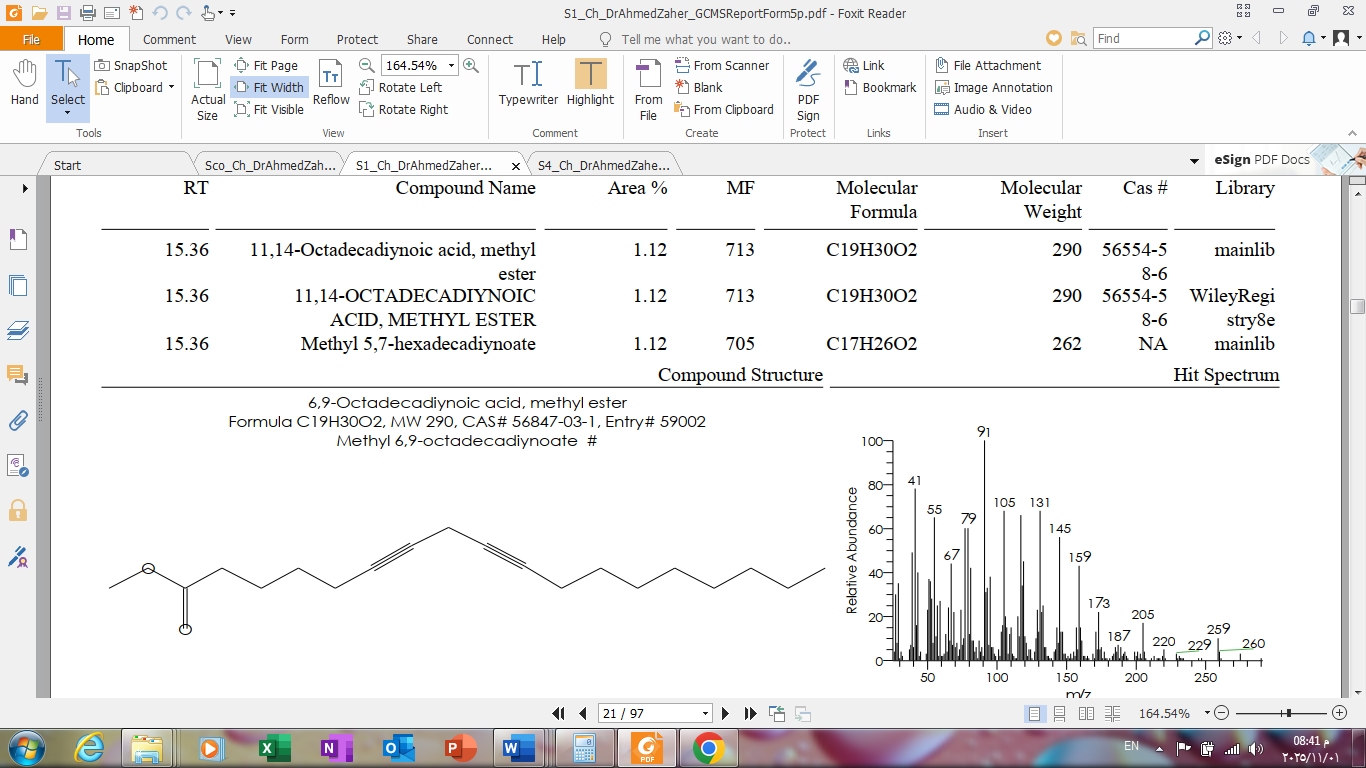


**Fig. S1** MS/MS fragmentation of some of the identified compounds by using GC-MS


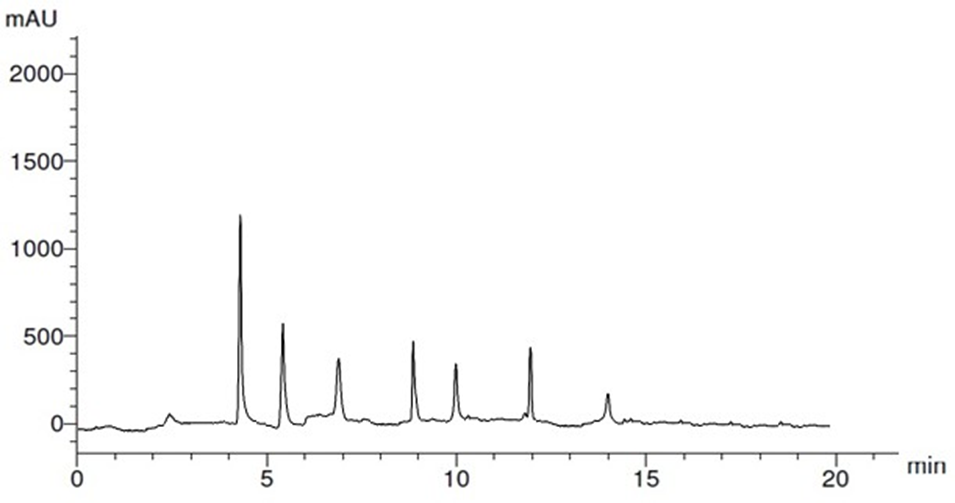


**A**


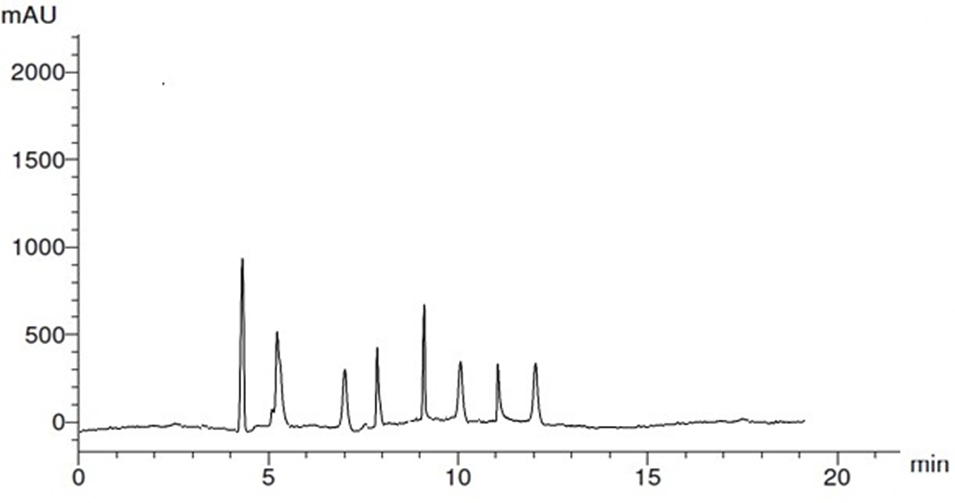


**B**

**R_t_ (min)**

**R_t_ (min)**

**Fig. S2** HPLC analysis of flavonoids in the ethyl acetate extract of *Adhatoda vasica* proliferated shoots (30-day-old)-treated cobalt (50 µM).

1. Chromatogram of the detected flavonoids in the ethyl acetate extract of *Adhatoda vasica* proliferated shoots.
2. Chromatogram and list of the standard flavonoids used in the experiment
